# Overexpression of *PheNAC3* from moso bamboo promotes leaf senescence and enhances abiotic stress tolerance in *Arabidopsis*

**DOI:** 10.7717/peerj.8716

**Published:** 2020-03-31

**Authors:** Lihua Xie, Miaomiao Cai, Xiangyu Li, Huifang Zheng, Yali Xie, Zhanchao Cheng, Yucong Bai, Juan Li, Shaohua Mu, Jian Gao

**Affiliations:** 1 Key Laboratory of Bamboo and Rattan Science and Technology, International Center for Bamboo and Rattan, State Forestry and Grassland Administration, Beijing, China; 2 Pingdingshan University, Pingdingshan, Henan, China

**Keywords:** NAC transcription factors, Senescence, Abiotic stress, Moso bamboo, Overexpression, Transgenic *Arabidopsis*, Transcriptional activity, Subcellular localization, NAM domain, Phylogenetic analysis

## Abstract

The NAC family is one of the largest transcription factor families unique to plants, which regulates the growth and development, biotic and abiotic stress responses, and maturation and senescence in plants. In this study, *PheNAC3*, a *NAC* gene, was isolated and characterized from moso bamboo (*Phyllostachys edulis*). PheNAC3 belong to the NAC1 subgroup and has a conserved NAC domain on the N-terminus, which with 88.74% similarity to ONAC011 protein. PheNAC3 localized in the nucleus and exhibited transactivation activity. *PheNAC3* was upregulated during the process of senescence of leaves and detected shoots. *PheNAC3* was also induced by ABA, MeJA, NaCl and darkness, but it had no remarkable response to PEG and SA treatments. Overexpression of *PheNAC3* could cause precocious senescence in *Arabidopsis*. Transgenic *Arabidopsis* displayed faster seed germination, better seedling growth, and a higher survival rate than the wild-type under salt or drought stress conditions. Moreover, *AtSAG12* associated with senescence and *AtRD29A* and *AtRD29b* related to ABA were upregulated by *PheNAC3* overexpression, but *AtCAB* was inhibited. These findings show that *PheNAC3* may participate in leaf senescence and play critical roles in the salt and drought stress response.

## Introduction

Moso bamboo (*Phyllostachys edulis*) is an important biological resource because its juvenile shoots are used for food and also in industrial production for its timber. It is widely distributed in China, accounting for more than 70% of the national bamboo forest area and has an important economic and ecological value. The flowering interval for moso bamboo is infrequent and ranges from 67 to 100 years. Flower and shoot degeneration of moso bamboo cause a huge economic and ecological loss and has a severe impact on the ecological environment (Liao & Huang, 1984). Shoot degeneration includes shoot apical meristem senescence and programed cell death. Therefore, it is important to determine the specific molecular mechanisms involved in the process of moso bamboo senescence.

Leaves, as photosynthetic organs, supply nutrients to the plant and are involved in plant senescence. In the process of senescence of leaves, macromolecules are degraded and senescence-related genes are upregulated or downregulated. Most transcription factors (TFs), including NAC (no apical meristem, Arabidopsis transcription activation factor 1 and 2, cup-shapedcotyledon 2), WRKY, bZIP (Basic region/leucine zipper motif), MYB (v-myb avian myeloblastosis viral oncogene homolog) and AP2/EREBP (APETALA2/ethylene-responsive element binding proteins), act as important regulators in the senescence process of leaves (Buchanan-Wollaston et al., 2005; Guo & Gan, 2012). However, there are few reports on the molecular mechanism of leaf senescence in moso bamboo.

NAC protein is a plant-specific transcription factor that not only participates in plant development and the stress response but also regulates the plant senescence process (Aida et al., 1997; Mao et al., 2010; Mitsuda et al., 2005; Liu, Sun & Wu, 2016; Guo, Cai & Gan, 2004; Kim, Chung & Woo, 2011; Christiansen & Gregersen, 2014). Several NAC genes related to senescence from different species were cloned and analyzed. For example, AtORE1, OsNAC2 and SIORE2 are members of the ORE1 subgroup of the NAC gene family, which regulate leaf senescence (Kim et al., 2009; Mao et al., 2017; Lira et al., 2017). AtNAP, GhNAP, OsNAP, SiNAP and BeNAP belong to the NAP subgroup of the NAC gene family, and all of which also regulate senescence (Guo & Gan, 2006; Chen et al., 2011; Liang et al., 2014; Fan et al., 2015; Ren et al., 2017a). Moreover, Most NAC genes regulate plant senescence by participating in the regulatory pathways of senescence-related hormones and/or stress response, such as abscisic acid (ABA), methyl jasmonate (MeJA), ethylene, salicylic acid (SA), drought, salt stress, dark and so on. OsNAC2 promotes leaf senescence via ABA biosynthesis and negatively regulates various abiotic stresses in rice (Luo et al., 2016). OsNAP confers an abiotic stress response through the ABA pathway (Chen et al., 2014). These results indicate that NAC homologous genes related to senescence of different species may have similar functions and are closely related to hormone or stress-induced senescence.

In *Arabidopsis*, AtNAC1, belonging to NAC1 subgroup, promotes lateral root development (Xie et al., 2001). In moso bamboo, the study of Wang et al. (2016) indicate that *PeNAC1* are targeted for degradation by *ped*-miR164b and shared a highly conserved N terminal DNA binding domain (94.52% homology with N terminal DNA binding domain of AtNAC1). The overexpression of *PeNAC1* may promote lateral root development and enhance tolerance to salinity and drought stress in *Arabidopsis* (Wang et al., 2016). In rice, as a member of NAC1 subgroup, ONAC011 acts as a negative regulator in drought tolerance of rice (Fang, Xie & Xiong, 2014). Moreover, ONAC011-overexpressing (OE) lines started heading 5–13 days earlier than control, and chlorophyl degradation of OE lines started at an early stage compared with wild-type, so that overexpression of ONAC011 can promote leaf senescence and accelerate heading time (Mannai et al., 2017). To some extent, the function of proteins which were classified into same subfamily might be both conserved and species-specific between moso bamboo and other plants.

For moso bamboo, based on the genome and sequencing, Peng et al. (2013) speculated that a potential connection between drought-responsive and flowering genes of moso bamboo and the NAC genes might be involved in flower senescence. The study of Li et al. (2015) divide the 125 candidate NAC TFs of moso bamboo into 17 subgroups and speculated that members of subgroups 1 and 14 might be involved in the senescence process of moso bamboo. Chen & Ding (2013) found that PeNAC1 had high similarity to AtNAP (involved in senescence regulation) in *Arabidopsis*. However, Li et al. (2015) and Chen & Ding (2013) did not conduct an in-depth analysis of their functions. Up to now, only the study of Li et al. (2019) indicates that PheNAP2 and PheNAP3 belong to NAP subgroup (which promote leaf senescence) and were involved in the meristem senescence process in harvested bamboo. The ectopic expression of *PheNAP2* and *PheNAP3* promotes leaf senescence in *Arabidopsis*. To the best of our knowledge, no further research has been conducted on the senescence-related NAC genes of moso bamboo.

In this study, to explore the senescence-related possible NAC TF, the homologous gene of *ONAC011* were analyzed through homologous alignment, and a NAC transcription factor named PheNAC3 was obtained. The amino acid (aa) sequences of PheNAC3 with 88.74% similarity to ONAC011 protein. And PheNAC3 possesses five more amino acids in the N-terminus of the protein sequence than that of PeNAC1 studied by Wang et al. (2016) (and possesses 15 additional bases at the 5′-end as compared to CDS of *PeNAC1*). *PheNAC3* was upregulated during the natural senescence of leaves and was induced by ABA and NaCl in detected leaves of moso bamboo. The overexpression of *PheNAC3* might promote the senescence process and increase abiotic stress resistance in *Arabidopsis*.

## Materials and Methods

### Plant material, growth conditions and treatments

The *Arabidopsis thaliana* ecotype Columbia-0 (Col-0), all transgenic plants, and moso bamboo (*Phyllostachys edulis*) seedlings were planted in a greenhouse under a 16/8 h light/dark cycle (long daylight) at 23 °C. The different developmental stages of moso bamboo leaves were sampled according to Ren et al. (2017b).

Treatments for senescence induction were performed with bamboo leaves and solutions of various substances. The third leaf from 2 to 3-month-old moso bamboo seedlings was detached and incubated in ABA solution (100 µM), MeJA solution (100 µM), SA solution (100 µM), ethrel (ETH) solution (600 mg/L), NaCl solution (200 mM), polyethylene glycol 6000 (PEG 6000) solution (20%, m/v), or sterile water. For dark treatment, the detached leaves were incubated in sterile water under darkness. The leaf samples were collected at 0, 1, 12, 24 and 48 h after treatment.

To detach Moso bamboo shoots, after the sheaths of the shoot tips emerged from the soil, they were manually removed after they reached the height of 35 ± 5 cm. The shoots were placed at room temperature, sampling at 0, 12, 24 and 48 h, respectively.

### Isolation of PheNAC3 and its sequence analysis

Using the protein sequence of ONAC011 (Os06g0675600) as a query to search against Bamboo.Hic.pep database (http://forestry.fafu.edu.cn/db/PhePacBio) (Wang et al., 2017; Zhao et al., 2018), two peptide sequences (PH02Gene43417.t1 and PH02Gene32306.t1) were found to have high similarities (over 83%) with that of ONAC011 protein, namely PheNAC2 and PheNAC3, respectively. PheNAC3 had higher similarities with ONAC011 than PheNAC2. According to the CDS sequence of *PheNAC3* gene retrieved from Bamboo.Hic.cds database (http://forestry.fafu.edu.cn/db/PhePacBio), Primer Premier 5 software was used to design the primers to isolate PheNAC3.

The genome sequence and the promoter fragments (from −2,000 bp to the initiation codon) of *PheNAC3* were retrieved from moso bamboo genome database (http://forestry.fafu.edu.cn/db/PhePacBio). The Gene Structure Display Server (http://gsds.cbi.pku.edu.cn/) was used to visualize the intron patterns (Hu et al., 2015). The conserved NAM domain was analyzed with the Conserved Domains Database (http://www.ncbi.nlm.nih.gov/Structure/cdd/cdd.shtml). Multiple sequence alignment and neighbor-joining phylogenetic analysis of PheNAC3 was performed with the program Clustal X 1.83 and MEGA 7 (1,000 bootstrap replicates). The *cis*-acting reglatory elements were identified with the online program Plant CARE (http://bioinformatics.psb.ugent.be/webtools/plantcare/html/).

The LOC ID of NAC family proteins used for constructing the phylogenetic tree are as follows: *Arabidopsis thaliana*, ATAF1 (ANAC002, AT1G01720.1), ORE1 (ANAC092, AT5G39610.1). ORS1 (ANAC059, AT3G29035.1), CUC1 (ANAC054, AT3G15170.1), CUC2 (ANAC098, AT5G53950.1), CUC3 (ANAC031, AT1G76420.1), NAC1 (ANAC022, AT1G56010.2), NAP (ANAC029, AT1G69490.1), JUB1 (ANAC042, AT2G43000.1), RD26 (ANAC072, AT4G27410.2), ANAC016 (AT1G34180.1), VNI2 (ANAC083, AT5G13180.1); *Oryza sativa*, ONAC011 (OMTN4, Os06g46270), ONAC104 (OMTN6, Os08g10080), ONAC060 (OMTN3, Os12g41680), ONAC002 (SNAC1, Os03g60080), ONAC058 (OsNAP, Os03g21060), ONAC131 (Os12g03040). ONAC002 (OsNAC2, Os04g38720); *Brachypodium distachyon*, BdNAC010 (Bradi1g32660), BdNAC054 (Bradi3g17287), BdNAC067 (Bradi4g02060), *Petunia hybrida*, StNAC262 (LOC102595632). The conserved motifs in the above senescence-related NAC sequences were defined by MEME version 4.12.0 (http://meme-suite.org/tools/meme) (Bailey et al., 2009).

### Gene expression analysis

Total RNA was extracted using RNAiso Plus (Code No.: 9108; Takara, Kusatsu, Japan) according to manufacturer’s instructions. First-strand cDNA was synthesized using a PrimeScript RT Reagent Kit with gDNA Eraser (Code No: RR420A; Takara, Kusatsu, Japan). The specific primers were designed using Primer 3 Input software (version 4.1.0). The quantitative PCR assays were performed as described by Cheng et al. (2017). The primers used for qRT-PCR are listed in Table S1. The tonoplast intrinsic protein 41 gene (*TIP41*) (Fan et al., 2013) and *ACT2* (Chen et al., 2011) were used as reference genes in moso bamboo and *Arabidopsis*, respectively.

### Subcellular localization and transcriptional activation

For the subcellular localization experiment, the full-length coding sequence of PheNAC3 without the stop codon was cloned and inserted into the PCAMBIA2300-35S-EGFP vector. The functional vectors of green fluorescent protein (GFP)-tagged PheNAC3 were introduced into *Agrobacterium tumefaciens* strain GV3101. The transformed *Agrobacterium* strain was infiltrated into the fully expanded leaves of 4-week-old tobacco (*Nicotiana benthamiana*). After 48 h, the GFP signal was detected by fluorescence microscope. The subcellular localization also was performed by transfecting GFP-tagged PheNAC3 into *Arabidopsis* protoplasts according to Yoo, Cho & Sheen (2007).

For transcriptional activation analysis in yeast, the full-length PheNAC3 cDNA was inserted into the PGBTK7 vector. The fusion vector of PheNAC3 and the GAL4 DNA-binding domain were transformed into yeast strain AH109 according to the manufacturer’s instructions (Clontech). The transactivation activity of the PheNAC3 protein was evaluated according to growth status and α-galactosidase activity (Chen et al., 2011; Chai et al., 2014).

### Overexpression and stress treatments

The full-length cDNA of PheNAC3 was cloned into the pCAMBIA 2300 vector according to Cui et al. (2013). The pCAMBIA 2300-PheNAC3 vector was introduced into *Arabidopsis* (Columbia-0, Col-0) through the floral dipping method (Clough & Bent, 1998). Putative transgenic plants were selected on 1/2 Murashige and Skoog (MS) medium solid plates supplemented with 50 mg/L kanamycin.

To observe the effects of NaCl or mannitol on seed germination and phenotypic differences between WT and OE-PheNAC3 plants, three independent PheNAC3 overexpressing lines (T_3_) and WT Arabidopsis plants were tested according to method previously methord (Hou et al., 2018). WT and PheNAC3 seeds were sown on 1/2 MS medium solid plates with 150 mM NaCl or 200 mM mannitol, stratified at 4 °C for 2 days and then transferred to long-day growth conditions (16 h light/8 h dark cycle at 23 ± 2 °C) in a growth chamber. Seed germination rates was daily were measured after 2 days until the number of germinated seeds no longer increased. The taproot lengths and lateral roots per taproot were measured after 10 days.

## Results

### *PheNAC3* is a homolog of *ONAC011* and *NAC1* in moso bamboo

**Figure 1:**
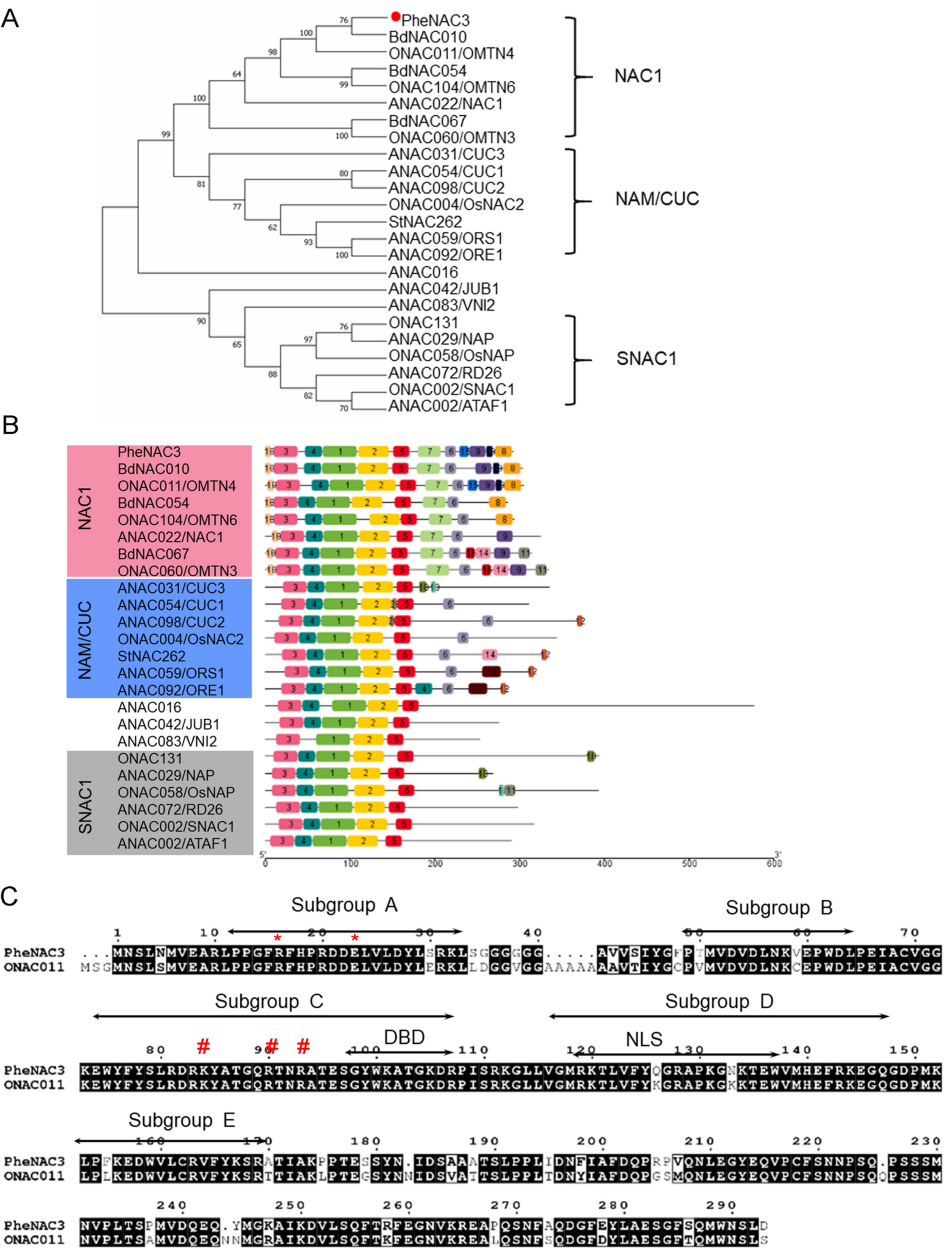
Sequence and phylogenetic analysis of PheNAC3. (A) Phylogenetic relationships among PheNAC3 proteins and 24 senescence-related NAC proteins. The numbers beside each node represent bootstrap values (≥50%) based on 1,000 replications. PheNAC3 is marked by a red circle. (B) Conserved motifs analyzed through MEME. Details of the individual motifs is shown in Fig. S2. (C) Two-sequence alignment of PheNAC3 and ONAC011. Arrows indicate the five conserved subdomains (A–E). The red asterisk denotes the residues (arginine and glutamic acid) forming a salt bridge stabilizing the dimerization interface; the red pound sign denotes the residues (lysine and arginine) in a region containing several highly conserved residues of importance to DNA binding.

To identify the NAC TFs associated with senescence in moso bamboo, the protein sequence of ONAC011 was used as a query to search against Bamboo.Hic.pep database (http://forestry.fafu.edu.cn/db/PhePacBio). One putative NAC protein (moso bamboo database: PH02Gene32306.t1; NCBI ID: FP095491) sharing high similarity (87%) with ONAC011 protein was named PheNAC3. Based on the analysis of intron–exon arrangement and conserved domain, the results showed that PheNAC3 contained two introns, three exons and the complete NAM domain (pfam02365; Fig. S1). Phylogenetic analysis of PheNAC3 and other NACs related to senescence revealed that PheNAC3 and ONAC011 were classified into one clade with 100% bootstrap support (Fig. 1A). Furthermore, the conserved motif distribution was consistent with the classification of the phylogenetic analysis (Fig. 1B).

In previous studies, most of the NAC proteins possessed A–E subdomains in the N-termini that conferred the DNA-binding activity. In this study, motifs 3, 4, 1, 2 and 5 specifying the NAM subdomain A–E were present in PheNAC3 proteins. In the C-termini, motif 6 was found in both NAC1 and NAM/CUC subgroups, while motifs 7, 8, 9 and 10 were only found in NAC1 subgroup. Moreover, motif 12 was only present in most of the members of subgroup NAM/CUC. Multiple sequence alignment showed that PheNAC3 showed with 88.74% similarity to ONAC011 protein (Fig. 1C). The PheNAC3 protein also had a conserved core sequence of DBD and NLS in subdomain C and D. Furthermore, sequence alignment indicated that the CDS sequence of PheNAC3 was longer than that of PeNAC1 by only 15 bases at the 5′ end. The amino acid encoded by the 15 base-sequence corresponded to motif 10 based on conserved motif analysis through MEME (Figs. S1B, S1 and S2).

### PheNAC3 was localized in the nucleus and possessed transcriptional activity

**Figure 2:**
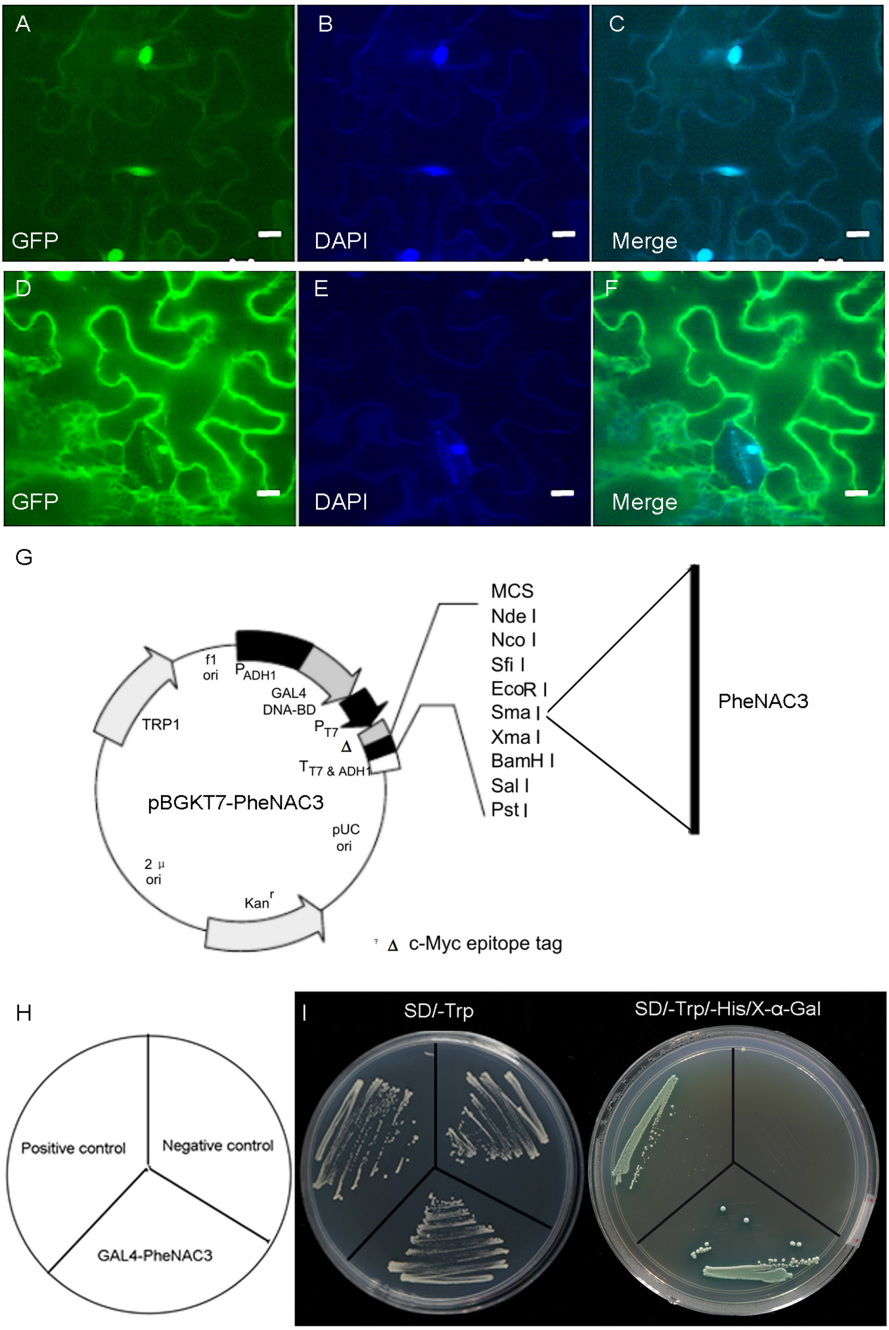
Subcellular localization and transcriptional activity of PheNAC3. (A–C) Nuclear localization of PheNAC3. (D–F) Subcellular localization of free GFP. (A and D) GFP, fluorescence of GhNAP-GFP fusion protein and free GFP protein, respectively; (B and E) DAPI, the protoplasts are stained with DAPI to visualize the nucleus; (C and F) Merge, merged images of GFP and DAPI ones; Bar = 10 μm. (G) Basal map for plasmid pGBKT7–PheNAC3. (H and I) Analysis of transcriptional activity for PheNAC3. The positive control: pGBKT7-53 + pGADT7-T, the negative control: the empty pGBKT7 vector, SD/–Trp and SD/–Trp/-His/X-α-Gal media for examination of growth and the expression of α-galactosidase.

A previous study reported that OMTN4/ONAC011 is a nuclear protein and putative transcriptional activator (Fang, Xie & Xiong, 2014). To determine the subcellular localization of PheNAC3, the PheNAC3-GFP fusion vector was transiently expressed in tobacco (*Nicotiana benthamiana*). The PheNAC3-GFP fusion protein was localized in the nucleus (Fig. 2A), as the GFP fluorescence in all cases colocalized with the fluorescence of the 4′,6-diamidino-2-phenylindole marker. The control GFP was distributed throughout the cell. In addition, the PheNAC3-GFP fusion protein also was localized in nucleus of *Arabidopsis* protoplasts (Fig. S3).

To examine whether PheNAC3 engages in transcriptional activation, the full-length PheNAC3 was fuzed to the GAL4 DBD of the pGBKT7 vector (Fig. 2B). The resulting constructs were expressed in AH109 yeast cells. The empty pGBKT7 vector was used as a negative control, and pGBKT7-53 and pGADT7-T were used as positive controls. All the transformants grew well on synthetic defined (SD)/-Trp medium, but only the yeast cells as a positive control and PheNAC3-BD could survive and simultaneously turn blue on SD/-Trp/-His/X-α-Gal medium (Fig. 2C).

### Expression profile of PheNAC3 during senescence of moso bamboo leaves and shoots

To evaluate the contribution of PheNAC3 in leaf senescence, we further analyzed the relative expression levels of PheNAC3 in moso bamboo through qRT-PCR. The results show that there were similar expression levels of PheNAC3 in roots and leaves (Fig. S4A). According to the photochemical efficiency of photosystem II (*Fv*/*Fm*) and the content of chlorophyl, the developmental stages of moso bamboo leaves were divided into young leaves, mature leaves, and senescent leaves (Ren et al., 2017b). There was an increase in *PheNAC3* transcript accumulation during the natural senescence of leaves (Fig. S4B).

According to the physiological and biochemical changes during the senescence of harvested shoots, sharp transitions occurred at 12 and 24 h, as compared to 0 h (Li et al., 2019). Additionally, the detached shoots become inedible after being stored for 24 h. Therefore, four representative storage time points (0, 12, 24 and 48 h) were selected to analyze the relative expression pattern of *PheNAC3*. The results showed that *PheNAC3* was significantly upregulated (approximately 3-fold higher than 0 h) at 12 h and continued to increase expression at 24 h (approximately 6-fold higher) and 48 h (approximately 17-fold higher) (Fig. S4C).

**Figure 3:**
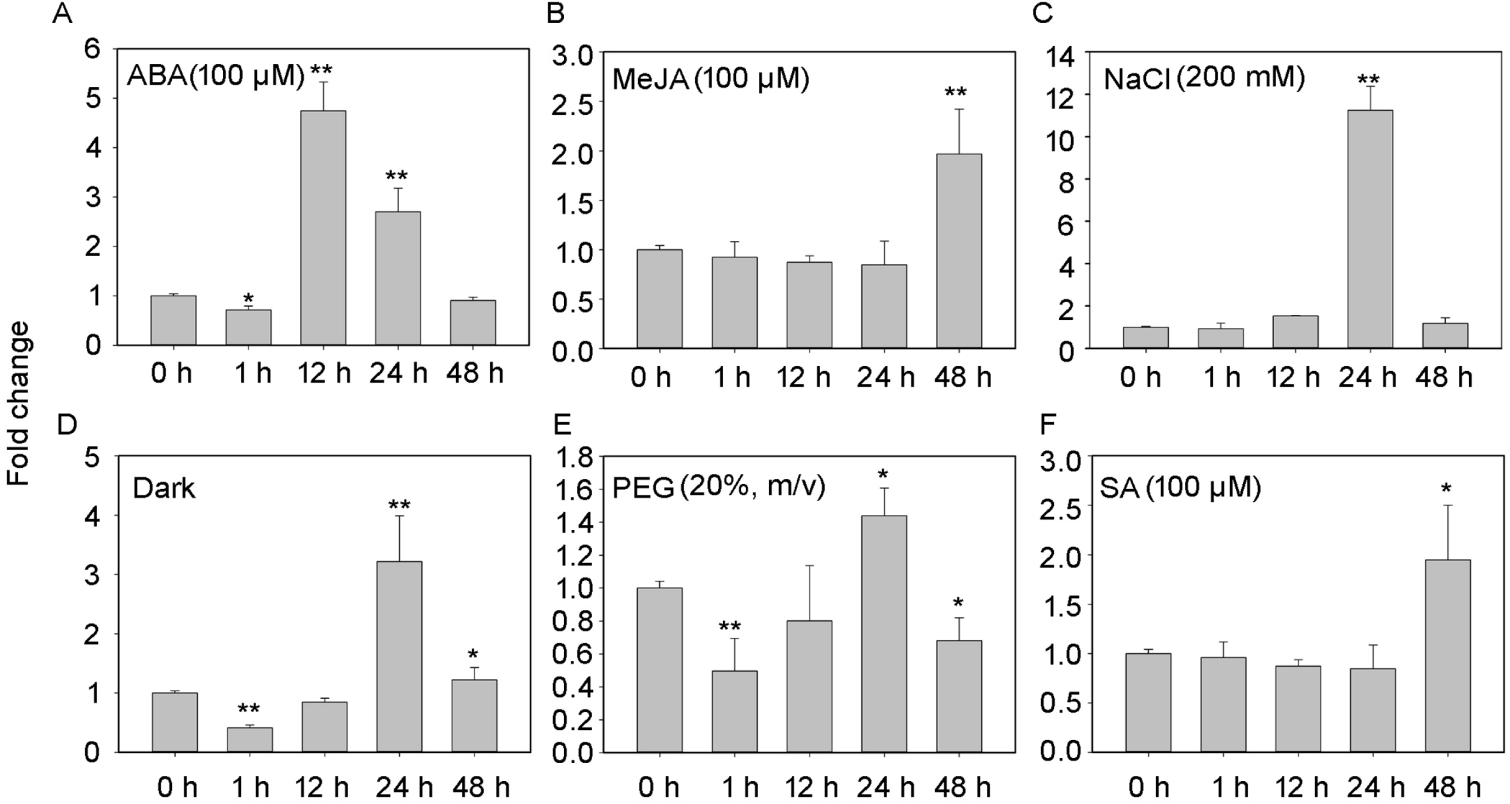
Expression analysis of *PheNAC3* via qRT-PCR during induced senescence. (A–F) Expression patterns of *PheNAC3* gene in detached leaves treated with ABA, MeJA, NaCl, Dark, PEG and SA; values represent the mean ± SE from three biological replicates. Statistically significant differences compared to 0 h (not-treated control) or young leaves are marked with asterisks (*P < 0.05 and **P < 0.01).

The transcript profiles of PheNAC3 were evaluated according to different treatments for detached leaves to induce senescence (i.e., treatment with ABA, MeJA, SA, darkness, salt, or drought; Fig. 3). After 48 h of treatments, all leaves turned yellow, and the chlorophyl content significantly decreased. Additionally, down- and upregulation of the chloroplast maintenance-related TF (*PheGLK1*, *PH01000738G0520*) and the senescence-associated gene (*PheSAG12*, *PH01001461G0020*) occurred, respectively (Fig. S3). PheNAC3 was upregulated during all seven senescence-induction treatments. Among these, PheNAC3 showed upregulated expression after ABA, darkness and salt stress. During the ABA treatment, the expression level of PheNAC3 was at least four-fold higher than that of the control within 12 h (Fig. 3A). The expression level of PheNAC3 was approximately 3-fold higher than that of the control after 24 h of darkness (Fig. 3D). Particularly, the expression level of PheNAC3 was approximately 11-fold higher than that of the control after 24 h of treatment with 200 mM NaCl (Fig. 3C). The distinct expression patterns of PheNAC3 suggested that it might function differently during different senescence processes.

### *PheNAC3* overexpression caused precocious leaf senescence phenotype

**Figure 4:**
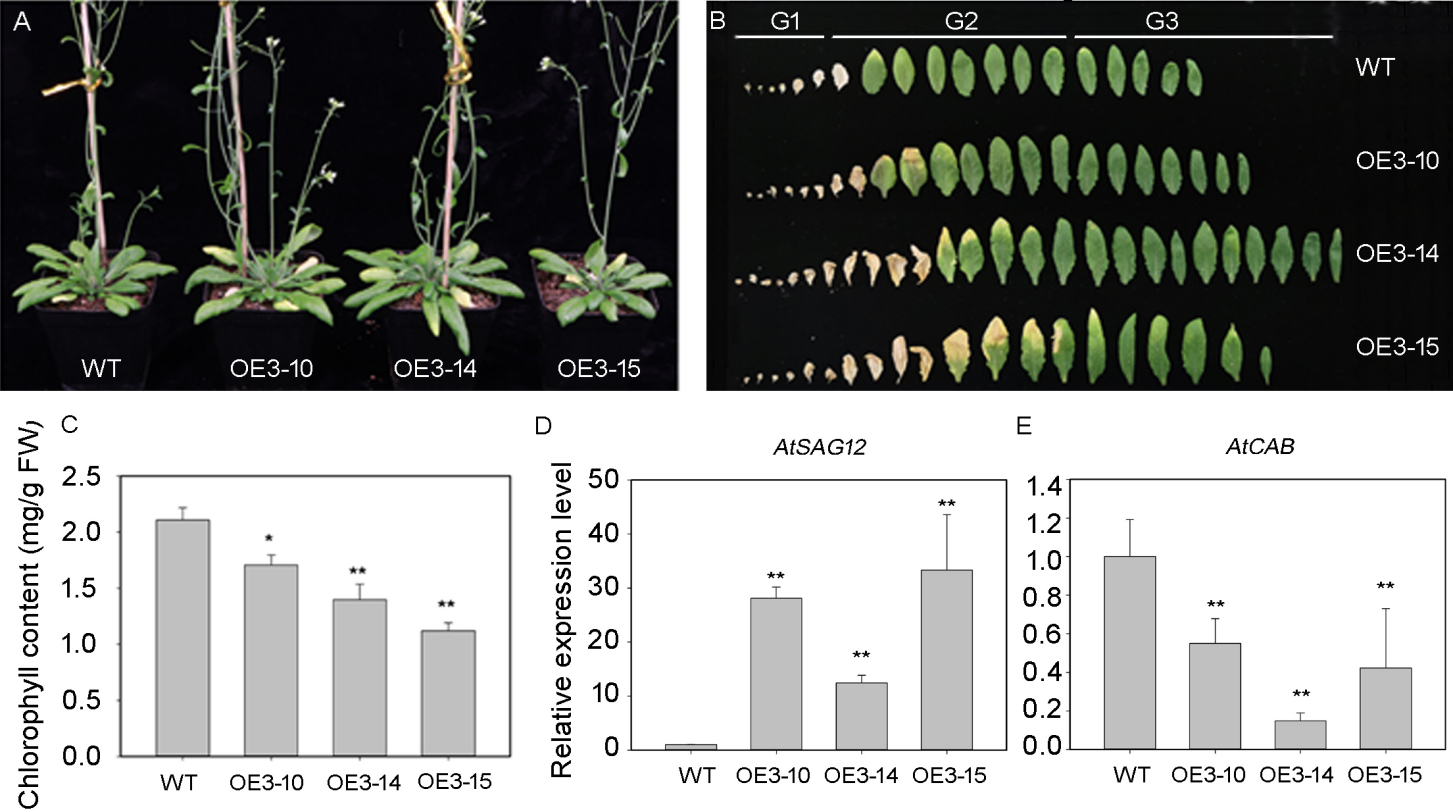
Ectopic expression of *PheNAC3* in *Arabidopsis* induced early leaf senescence. (A) The phenotype of 5-week-old Col and OE-PheNAC3 plants. (B) Leaves detached from plants in (A). G1–G3, three groups of leaves divided according to the senescence status and the method of Fan et al. (2015). G1, the first to the sixth leaves which were dry. G2, Leaf yellowing of OE3-15 lines started at an early stage compared with OE3-10 lines, OE3-14 lines and wild-type. Moreover, after 5 weeks of growth, the 13 or 14 leaves of OE3-15 lines started to turn yellow from tip of leaf. So, the seventh to the 14 leaves which were withered or turned yellow from the tip of leaf were classfied into G2 group. G3, the 15th to the last rosette leaves were G3 group. (C) Chlorophyll content in leaves from 5-week-old plants in (A) and (B). (D and E) Relative expression of two senescence marker genes, including SAG12 and CAB1, in G2 group of 5-week-old WT and OE-PheNAC3 plants.WT, wild-type *Arabidopsis*; OE3-10, OE3-14 and OE3-15, three independent lines. All experiments were repeated three times, with similar results. Asterisks indicate the significant difference between WT and OE-PheNAC3 (*P < 0.05 and **P < 0.01); error bars indicate the ± SE.

The PheNAC3 coding sequence, driven by the 35S promoter, was introduced into the wild-type (Col-0) plants to overexpress PheNAC3. After 3 weeks of growth, the PheNAC3 overexpressors (OE-PheNAC3) displayed the senescence phenotype (the tip of 7th leaf begins to turn yellow), while the non-yellowing phenotype was found in Col-0 (Fig. S6). After 5 weeks, both OE-PheNAC3 and Col-0 displayed the senescence phenotype (Fig. 4A). The expression level of PheNAC3 in OE-PheNAC3 lines was confirmed by RT-PCR (Fig. S7). The detached leaves were divided into three groups (G1–G3; Fig. 4B) according to the senescent condition. G2 and G3 were used to analyze the chlorophyl content and target gene expression levels. Compared with Col-0, the OE-PheNAC3 lines showed much lower chlorophyl content in G2 leaves (Fig. 4C; Fig. S8). The above results demonstrated that overexpression of PheNAC3 could induce leaf senescence-related physiological changes in *Arabidopsis*.

The expression profiles of senescence-specific marker genes *AtSAG12* and *AtCAB1* were analyzed with qRT-PCR during leaf senescence between OE-PheNAC3 and Col-0 plants (Figs. 4D and 4E). The results showed that *AtSAG12* was upregulated and *AtCAB1* was downregulated compared with Col-0. These results suggested that PheNAC3 acted as a positive regulator of leaf senescence.

Based on the analysis of the promoter region of PheNAC3 in PlantCARE, we found that seven ABRE *cis*-elements were contained in this region (Table S2). The relative expression level of PheNAC3 was upregulated (over 4 fold) in detected leaves of moso bamboo after ABA treatment. Furthermore, *AtNCED3*, *AtRD29A* and *AtRD29B* related to ABA biosynthesis and/or response genes were obviously upregulated in OE-PheNAC3 lines compared with those in Col-0 under normal conditions (Fig. S9).

### *PheNAC3* overexpression enhanced drought and salt tolerance of *Arabidopsis*

**Figure 5:**
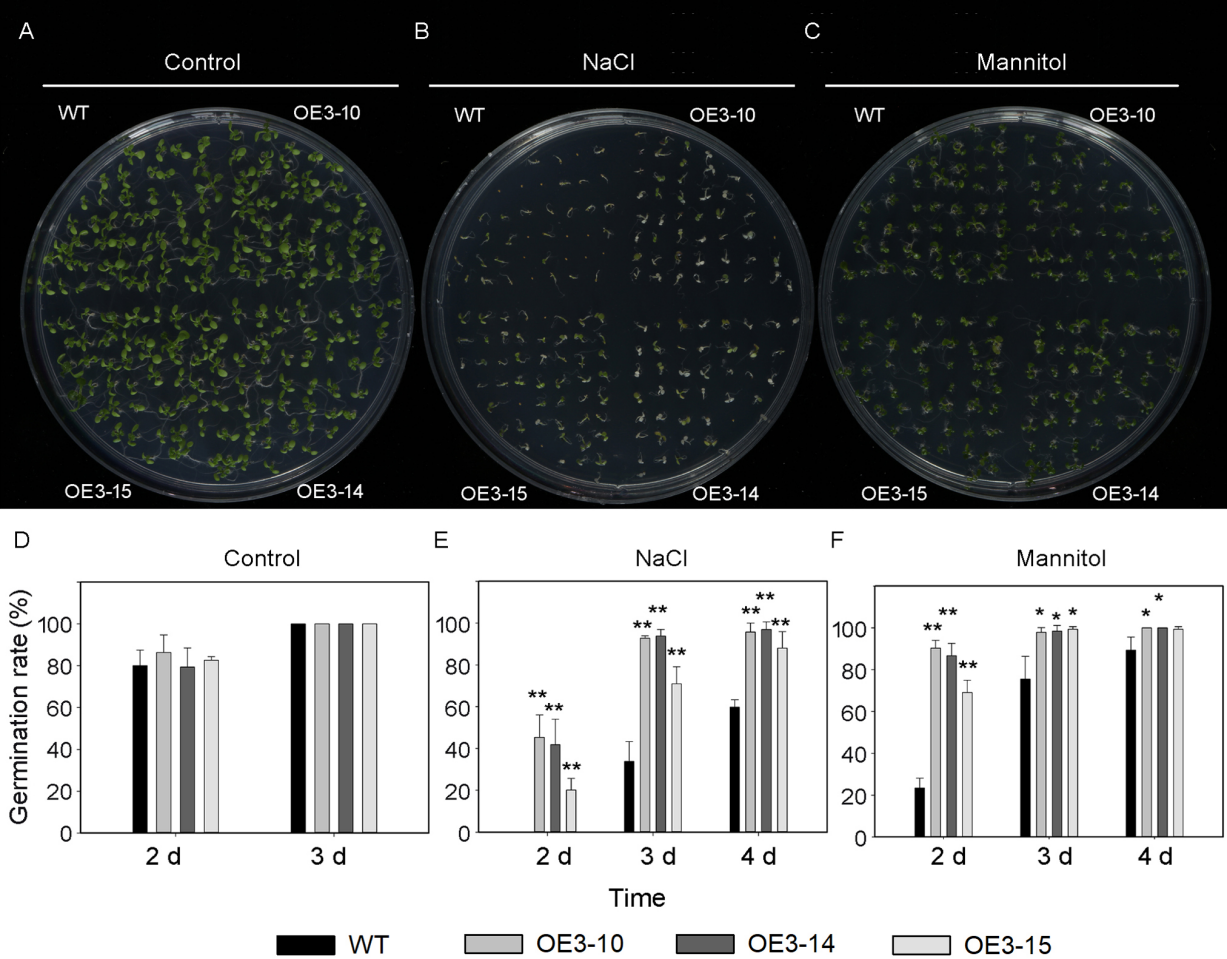
Seed germination of wild-type and *PheNAC3*-overexpressing *Arabidopsis* under mannitol or NaCl stress. (A–C) Seeds were germinated on 1/2 MS agar plates with or without mannitol or NaCl; photographs were taken 10 days after mannitol or NaCl treatment. (D–F) The germination rate was determined at 2–4 days after mannitol or NaCl treatment. (A and D) 1/2 MS agar plates; (B and E) 1/2 MS agar plates + 150 mM NaCl; (C and F) 1/2 MS agar plates + 200 mM Mannitol. WT, wild-type *Arabidopsis*; OE3-10, OE3-14 and OE3-15, three independent lines. The experiments were repeated three times with similar results. Asterisks indicate the significant difference between WT and OE-PheNAC3 (*P < 0.05 and **P < 0.01); error bars indicate the ± SE.

**Figure 6:**
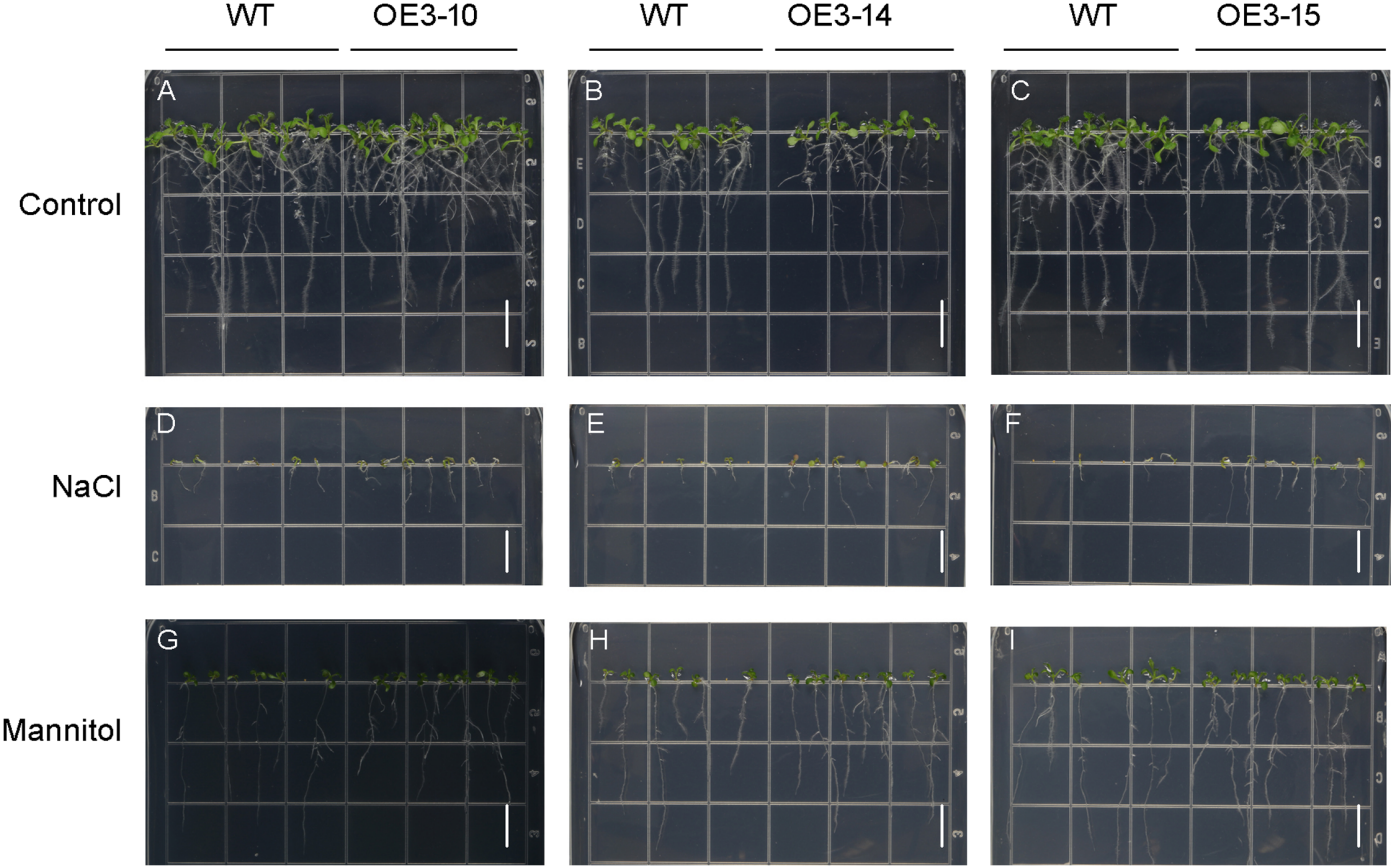
Phenotypic analysis of roots of wild-type (WT) and *PheNAC3*-overexpressing *Arabidopsis* under mannitol or NaCl stress. (A–C) 1/2 MS agar plates; (D–F) 1/2 MS agar plates + 150 mM NaCl; (G–I) 1/2 MS agar plates + 200 mM Mannitol. WT, wild-type *Arabidopsis*; OE3-10, OE3-14 and OE3-15, three independent lines. Photographs were taken 10 days after mannitol or NaCl treatment. Bar = 1 cm.

*ONAC011*/*OMTN4* may negatively regulate drought tolerance of rice (Fang, Xie & Xiong, 2014). In order to examine whether *PheNAC3* affects drought and salt tolerance in transgenic *Arabidopsis*, we examined the germination rate and growth of Col-0 and OE-PheNAC3 lines after 200 mM mannitol and 150 mM NaCl stress treatments (Figs. 5 and 6). The results showed that 3 days after germination, there was no significant difference in the germination rate (100%) and root length between wild-type and transgenic lines on 1/2 MS (Figs. 5 and 6; Fig. S10). On 1/2 MS with 150 mM NaCl, the germination rate of both Col-0 and OE-PheNAC3 lines was obviously decreased. However, the germination rate and root length of OE-PheNAC3 were significantly higher than that of wild-type (Figs. 5 and 6; Fig. S10). Approximately 90% of OE-PheNAC3 seeds germinated after 4 days, but only 60% of wild-type seeds germinated (Fig. 5). After 10 days, the average length of taproot of OE-PheNAC3 was greater than 0.79 ± 0.05 cm, while the WT was 0.27 ± 0.08 cm (Fig. 6; Fig. S10). At the same time, germination tests were also performed in the presence of 200 mM mannitol. The germination rates of OE-PheNAC3 were significantly higher than that of wild-type on the 2 and 3 days. The number of lateral roots were also significantly higher than those of the wild-type. After 10 days. The average numbers of lateral roots per in taproot of were OE-PheNAC3 and WT over 4.38 ± 0.46 and 1.71 ± 0.65, respectively, *p* < 0.004 (Fig. 6; Fig. S10). These results provided evidence that PheNAC3 positively regulates drought and salt resistance in *Arabidopsis*.

## Discussion

### PheNAC3 is a candidate senescence-related gene

NAC TFs play important and multiple roles in plant development, stress response and senescence regulation (He et al., 2005; Kim et al., 2009; Nakashima et al., 2012; Nuruzzaman, Sharoni & Kikuchi, 2013). In *Arabidopsis*, *AtNAC2* is involved in the salt stress response, lateral root development, and senescence regulation (He et al., 2005; Balazadeh et al., 2010). *AtNAP* negatively regulated the salt stress response and leaf senescence (Guo & Gan, 2006; Seok et al., 2017). In rice, *OsNAC2* and *OsNAP* exhibited a higher degree of sequence similarity to *AtNAC2* and *AtNAP*, respectively (Chen & Ding, 2013; Liang et al., 2014; Mao et al., 2017). *OsNAC2* is involved in leaf senescence regulation and negatively regulates rice abiotic stress reactions (Luo et al., 2016; Mao et al., 2017). OsNAP can increase the resistance to high salinity, drought, and low temperature stress and promote leaf senescence (Chen & Ding, 2013; Liang et al., 2014). Thus, it is generally acknowledged that homologous proteins with similar domains among different species may have the same or similar functions.

*ONAC011*/*OMTN4* is upregulated during natural senescence, which can promote leaf senescence and accelerate heading time in rice (Mannai et al., 2017). Furthermore, ONAC011 was strikingly reduced under drought stress conditions and increased under high salinity stress, and exhibited negative drought stress tolerance (Fang, Xie & Xiong, 2014).

Moso bamboo, as a perennial, lignified, and herbaceous plant, has great ecological, economic, and cultural value. There is a lack of systematic research on the mechanism of senescence regulation in moso bamboo because of the plant’s longevity. Thus, our study was focused on moso bamboo in order to find a novel NAC member of moso bamboo that can regulate senescence.

In this study, a senescence-induced NAC gene, was isolated from the senescent leaf cDNA library of moso bamboo and subsequently named *PheNAC3*. Sequence analysis revealed that *PheNAC3* encoded an NAC1-like NAC protein. The amino acid sequence of PheNAC3 protein is 88.74% similar to that ONAC011 protein. Based on transient expression and transcriptional activation analysis, PheNAC3 was determined to be a nuclear protein and exhibited transcriptional activation activity in yeast. These data indicated that PheNAC3 might function as a transcription factor and had a function similar to that of ONAC011.

### PheNAC3 participates in multiple senescence processes

To explore the function of PheNAC3 in senescence regulation, its expression pattern was initially analyzed during natural senescence, after treatment with ABA, MeJA, SA, NaCl and PEG, and during dark-induced leaf senescence. During natural leaf senescence, accumulation of PheNAC3 mRNA levels was similar to the relative expression pattern of ONAC011, which demonstrated that PheNAC3 could respond to senescence signals of age-dependent pathways (Mannai et al., 2017). Expression of PheNAC3 was significantly upregulated during ABA and NaCl treatments (Fig. 3). In addition, the promoter of PheNAC3 contained seven ABA-responsive elements (ABREs) and three stress-related elements (MBS: MYB drought inducibility; STRE: activation by heat shock, osmotic stress, low pH and nutrient starvation) (Table S2). These results indicated that PheNAC3 might be involved in the ABA-senTF module and NaCl-senTF module. At the same time, PheNAC3 was also upregulated during post-harvest senescence of bamboo shoots. The transcript level was positively correlated with the rate of loss of moisture, peroxidase activity, and catalase activity, and negatively correlated with lipoxygenase activity and phenylalanine ammonia-lyse activity (Li et al., 2019). Therefore, PheNAC3 participates in the regulation of post-harvest senescence of bamboo shoots. Taken together, these results suggest that PheNAC3 might be a senescence regulator during multiple senescence processes of moso bamboo.

### Ectopic expression of PheNAC3 can promote leaf senescence of *Arabidopsis*

In rice, ONAC011 was previously identified as a senescence regulator (Mannai et al., 2017). Here, the ectopic overexpression of *PheNAC3* triggered premature senescence of leaves in *Arabidopsis*. In *Arabidopsis*, *AtSAG12* and *AtCAB* were considered to be senescence marker genes (Lohman et al., 1994; Buchanan-Wollaston, 1994; Hensel et al. 1993) that were upregulated and downregulated during leaf senescence, respectively. In this study, the expression of two marker genes was upregulated and downregulated in OE-PheNAC3 transgenic *Arabidopsis*, respectively. This leads us to suspect that PheNAC3 promotes natural leaf senescence by regulating *AtSAG12* and *AtCAB* in *Arabidopsis*.

Our results showed that ABA promoted senescence of moso bamboo detached leaves, and PheNAC3 upregulated the expression during the senescence process. NCED3 is known as a key enzyme in ABA biosynthesis (Ren et al., 2007), and overexpression of NCED increases the ABA content (Tan et al., 1997; Hwang et al., 2010). In OE-PheNAC3 transgenic *Arabidopsis*, *AtNCED3* was upregulated. *AtABF4*, *AtRD29A* and *AtRD29B*, as ABA response genes, are upregulated by ABA (Yoshida et al., 2010; Cheong et al., 2010; Jia et al., 2012). Among them, the regulation of *AtRD29B* expression is only via the ABA-dependent pathway (Jia et al., 2012). In OE-PheNAC3 transgenic *Arabidopsis*, *AtRD29A* and *AtRD29B* were upregulated in leaves of G2 stage. These results suggested *PheNAC3* might promote leaf senescence via ABA biosynthesis.

### Ectopic expression of PheNAC3 can enhance abiotic stress tolerance in *Arabidopsis*

ONAC011 negatively regulates drought tolerance in rice (Mannai et al., 2017). PeNAC1 may promote lateral root development and enhance tolerance to salinity and drought stress (Wang et al., 2016). In our study, there was no significant difference in the growth, root length, or number of lateral roots of *PheNAC3* overexpressing in plants on 1/2 MS medium compared to wild-type *Arabidopsis*. This result is different from that of Wang et al. (2016), which indicates that it is caused by the difference in the motif10 in the N-terminus.

*RD29A* and *RD29B* were most frequently selected as stress-related downstream markers in *Arabidopsis* (Xiong et al., 2002). *RD29A* is an abiotic stress-responsive gene (Yamaguchi-Shinozaki et al., 1992; Yamaguchi-Shinozaki & Shinozaki, 1994) and enhanced the salt tolerance of transgenic tobacco and *Sophora japonica* (Yamaguchi-Shinozaki & Shinozaki, 1994; Wang et al., 2009). *RD29B* was upregulated in transgenic *Arabidopsis* with *GmProT1*, *GmProT2* and *MYB15*, which increases tolerance to drought and/or salt (Ding et al., 2009; Guo et al., 2016). In this study, the tolerance to drought and salt of OE-PheNAC3 transgenic *Arabidopsis* was increased, and the function of OE-PheNAC3 was similar to that of PeNAC1 (Wang et al., 2016). Additionally, the expression levels of RD29A and RD29B were upregulated in OE-PheNAC3 transgenic *Arabidopsis* compared with wild-type *Arabidopsis*. Therefore, the overexpression of PheNAC3 can enhance the abiotic stress tolerance of *Arabidopsis*.

## Conclusions

The protein sequences of PheNAC3 and ONAC011 have high similarity and belong to the NAC1 subfamily. PheNAC3 is determined to be a nuclear protein and exhibites transcriptional activation activity in yeas, indicating that PheNAC3 might function as a transcription factor. PheNAC3 is involved in the regulation of leaf senescence of moso bamboo. Ectopic expression of PheNAC3 can promote leaf senescence and enhance abiotic stress tolerance in *Arabidopsis*. In addition, the functional difference between PheNAC3 and PeNAC1 may be due to the fact that 15 additional bases at the 5′-end of PheNAC3 are different from the CDS of PeNAC1 Our results suggest that PheNAC3 play the multifaceted role in regulation of leaf senescence and abiotic stress responses.

## Supplemental Information

10.7717/peerj.8716/supp-1 Supplemental Information 1 Sequence analysis of PheNAC3. (A) The Intron/Exon structure of PheNAC3. (B) The two sequence alignment between PheNAC3 and PeNAC1. (C) NAM domain of PheNAC3 and PeNAC1.

10.7717/peerj.8716/supp-2 Supplemental Information 2 Details of the individual motifs obtained by MEME.

10.7717/peerj.8716/supp-3 Supplemental Information 3 Subcellular localization of PheNAC3 in *Arabidopsis* protoplast. (A) GFP, fluorescence of PheNAC3-GFP; (B) Bright, (C) Merge, merged images of GFP and Bright one, Bar = 50 μm.

10.7717/peerj.8716/supp-4 Supplemental Information 4 Expression analysis of PheNAC3 via qRT-PCR. (A) Expression analysis of *PheNAC3* in root, stem, and leaf. (B) Expression pattern of *PheNAC3* in leaves of 3-year-old seedlings. (C) Expression pattern of *PheNAC3* in the detached shoot.

10.7717/peerj.8716/supp-5 Supplemental Information 5 Chlorophyll and senescence marker transcript levels after 48 h of senescence-inducing treatments. (A) Images of the leaves after 48 h of senescence-inducing treatments. (B) Heat map indicating the relative transcript ratio of PheGLK1 (*PH01000738G0520*) and PheSAG12 (*PH01001461G0020*) normalized against values from samples harvested 0 and 48 h after ethylene treatment, respectively. Values represent the mean from at least three biological replicates. Black color = not detected. Statistically significant differences compared with the corresponding controls (*P* < 0.05) are colored accordingly to the scale. (C) Chlorophyll content in leaf samples. Values represent the mean ± SE from at least three biological replicates. Statistically significant differences compared to not-treated control are marked with asterisks (*P* < 0.05).

10.7717/peerj.8716/supp-6 Supplemental Information 6 The phenotype of 3-week-old Col and OE-PheNAC3 plants. WT, wild-type *Arabidopsis*. OE3-10, OE3-14 and OE3-15, three independent lines.

10.7717/peerj.8716/supp-7 Supplemental Information 7 The expression level of *PheNAC3* in OE-PheNAC3 lines was confirmed by RT-PCR. WT, wild-type *Arabidopsis*. OE3-10, OE3-14 and OE3-15, three independent lines.

10.7717/peerj.8716/supp-8 Supplemental Information 8 Relative expression of *AtSAG12* and *AtCAB1* genes in leaves of G3 in 5-week-old plants. WT: wild-type *Arabidopsis*. OE3-10, OE3-14 and OE3-15: three independent lines. All experiments were repeated three times. Asterisks indicate the significant difference between WT and OE-PheNAC3 (*P < 0.05, **P < 0.01); error bars indicate the ± SE.

10.7717/peerj.8716/supp-9 Supplemental Information 9 The relative expression of ABA-related genes in OE-PheNAC3 lines. (A–D) The expression level of NCED3, ABF4, RD29A, and RD29B, in G2 of 5-week-old WT and OE-PheNAC3 plants. (E–H) the four genes in G3 of WT, wild-type *Arabidopsis*. OE3-10, OE3-14 and OE3-15, three independent lines. All experiments were repeated three times, with similar results. Asterisks indicate the significant difference between WT and OE-PheNAC3 (*P < 0.05, **P < 0.01); error bars indicate the ± SE.

10.7717/peerj.8716/supp-10 Supplemental Information 10 Statistical analysis of root length and lateral root number of wild-type (WT) and *PheNAC3*-overexpressing *Arabidopsis* under mannitol or NaCl stress. (A–C) Statistical analysis of taproot length of wild-type (WT) and *PheNAC3*-overexpressing *Arabidopsis* under mannitol or NaCl stress. (D) Statistical analysis of lateral root number of wild-type (WT) and *PheNAC3*-overexpressing *Arabidopsis* under mannitol. WT, wild-type *Arabidopsis*. OE3-10, OE3-14 and OE3-15, three independent lines. All experiments were repeated three times. Asterisks indicate the significant difference between WT and OE-PheNAC3 (*P < 0.05, **P < 0.01); error bars indicate the ± SE.

10.7717/peerj.8716/supp-11 Supplemental Information 11 The list for primers.

10.7717/peerj.8716/supp-12 Supplemental Information 12 Hormone and stress related *cis*-elements in promoters of *PheNAC3*.

10.7717/peerj.8716/supp-13 Supplemental Information 13 Raw data.

## Additional Information and Declarations

### Data Availability

The following information was supplied regarding data availability: The raw measurements are available in the Supplemental Files. The protein sequence ONAC011 (Os06g0675600) is available at http://forestry.fafu.edu.cn/db/PhePacBio/download.php (Bamboo.Hic.pep.gz).
