# Supplementary figures and images for: Overexpression of PheNAC3 from moso bamboo promotes leaf senescence and enhances abiotic stress tolerance in Arabidopsis
Source: PeerJ. 2020 Mar 31;8:e8716. doi: 10.7717/peerj.8716 (PMC7120055; doi:10.7717/peerj.8716)

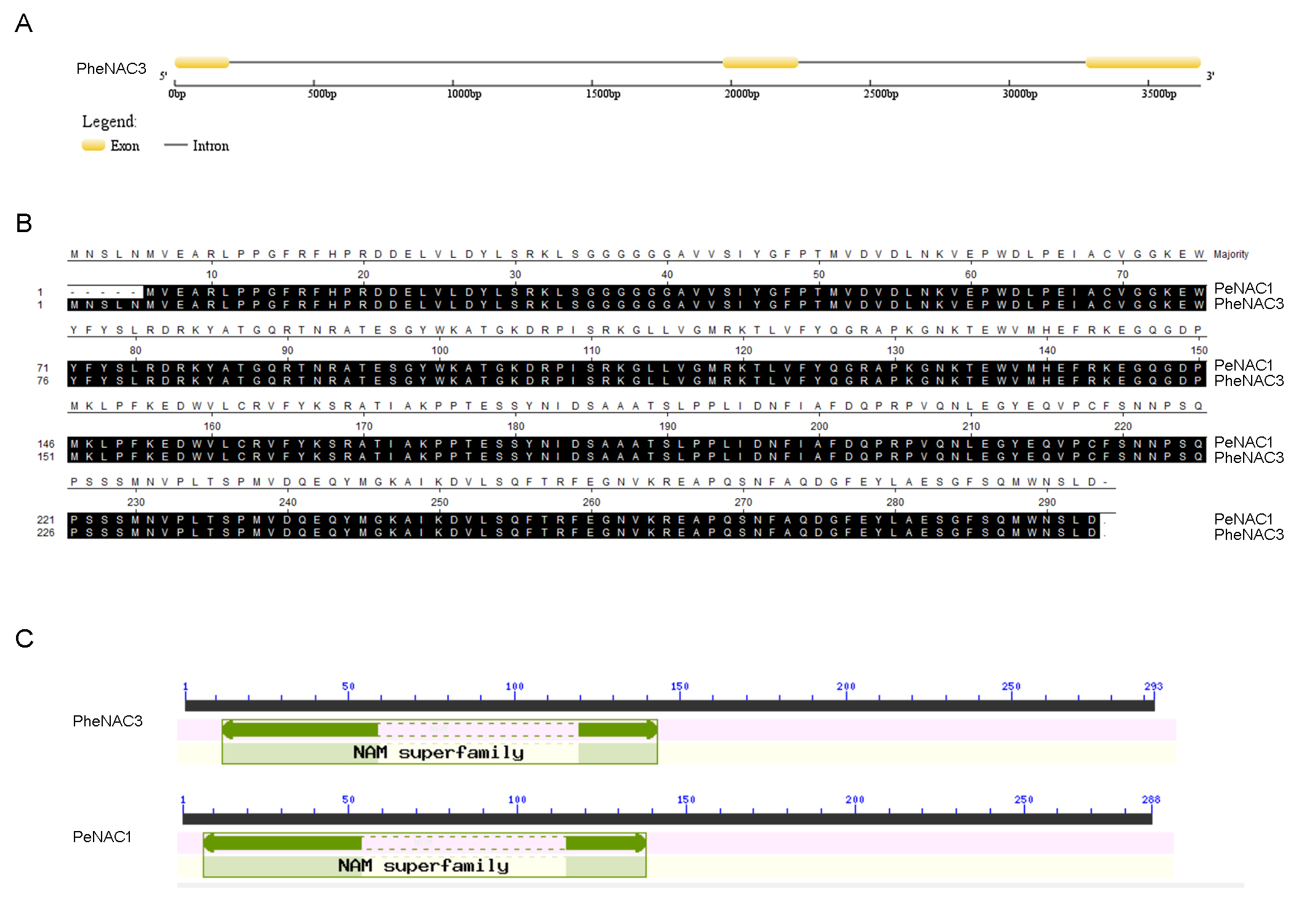

Supplement: Supplemental Information 1 — (A) The Intron/Exon structure of PheNAC3. (B) The two sequence alignment between PheNAC3 and PeNAC1. (C) NAM domain of PheNAC3 and PeNAC1. [file peerj-08-8716-s001.png]

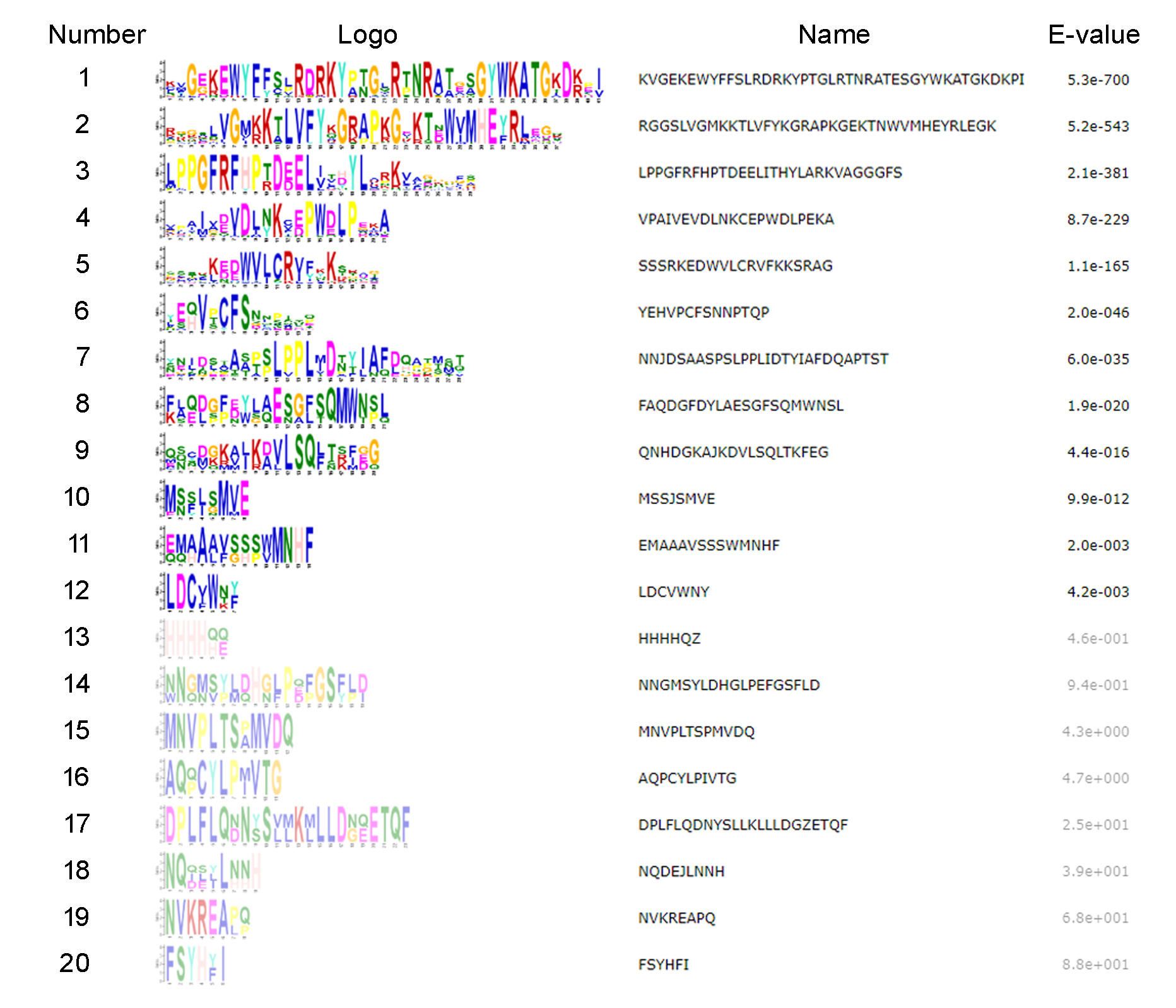

Supplement: Supplemental Information 2 [file peerj-08-8716-s002.png]

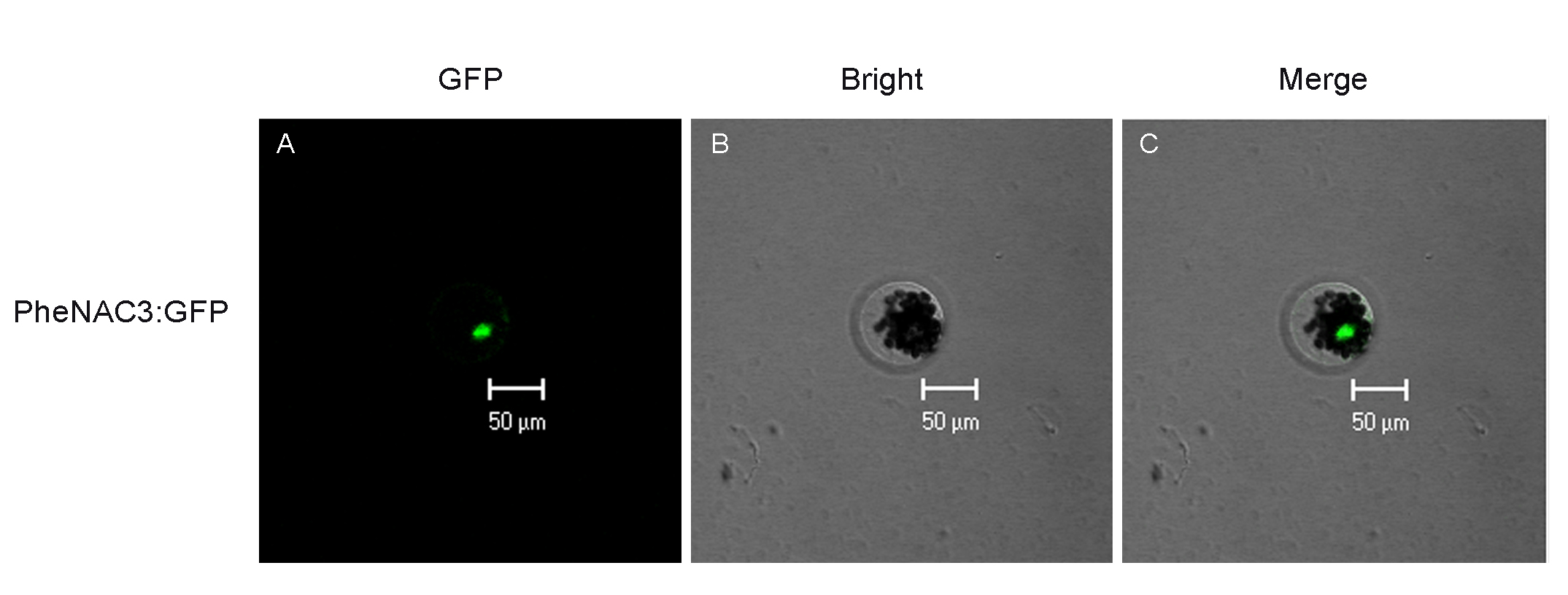

Supplement: Supplemental Information 3 — (A) GFP, fluorescence of PheNAC3-GFP; (B) Bright, (C) Merge, merged images of GFP and Bright one, Bar = 50 μm. [file peerj-08-8716-s003.png]

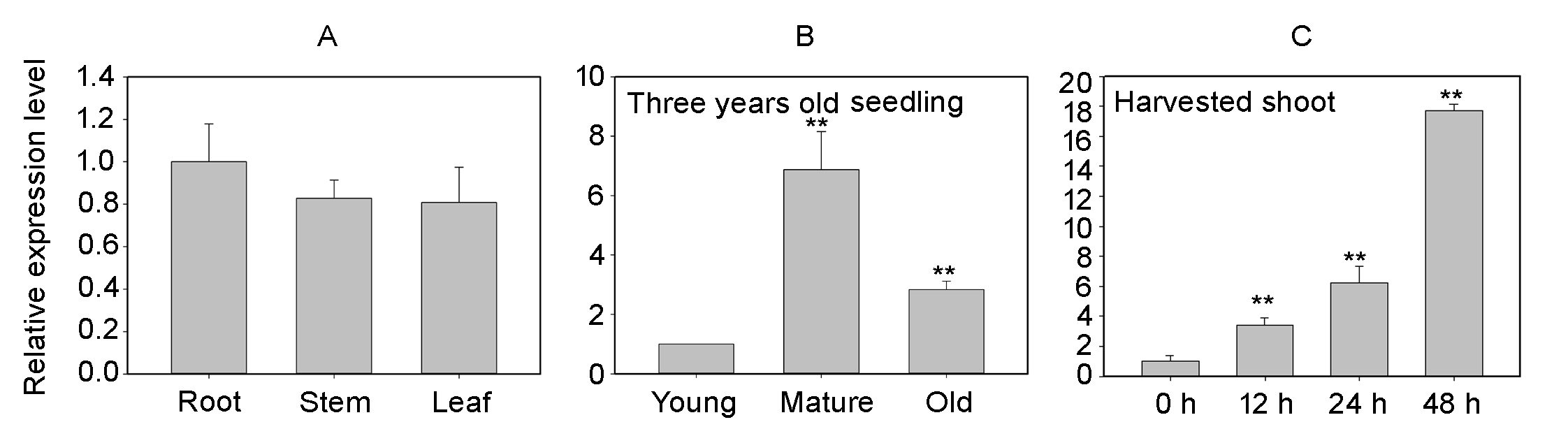

Supplement: Supplemental Information 4 — (A) Expression analysis of PheNAC3 in root, stem, and leaf. (B) Expression pattern of PheNAC3 in leaves of 3-year-old seedlings. (C) Expression pattern of PheNAC3 in the detached shoot. [file peerj-08-8716-s004.png]

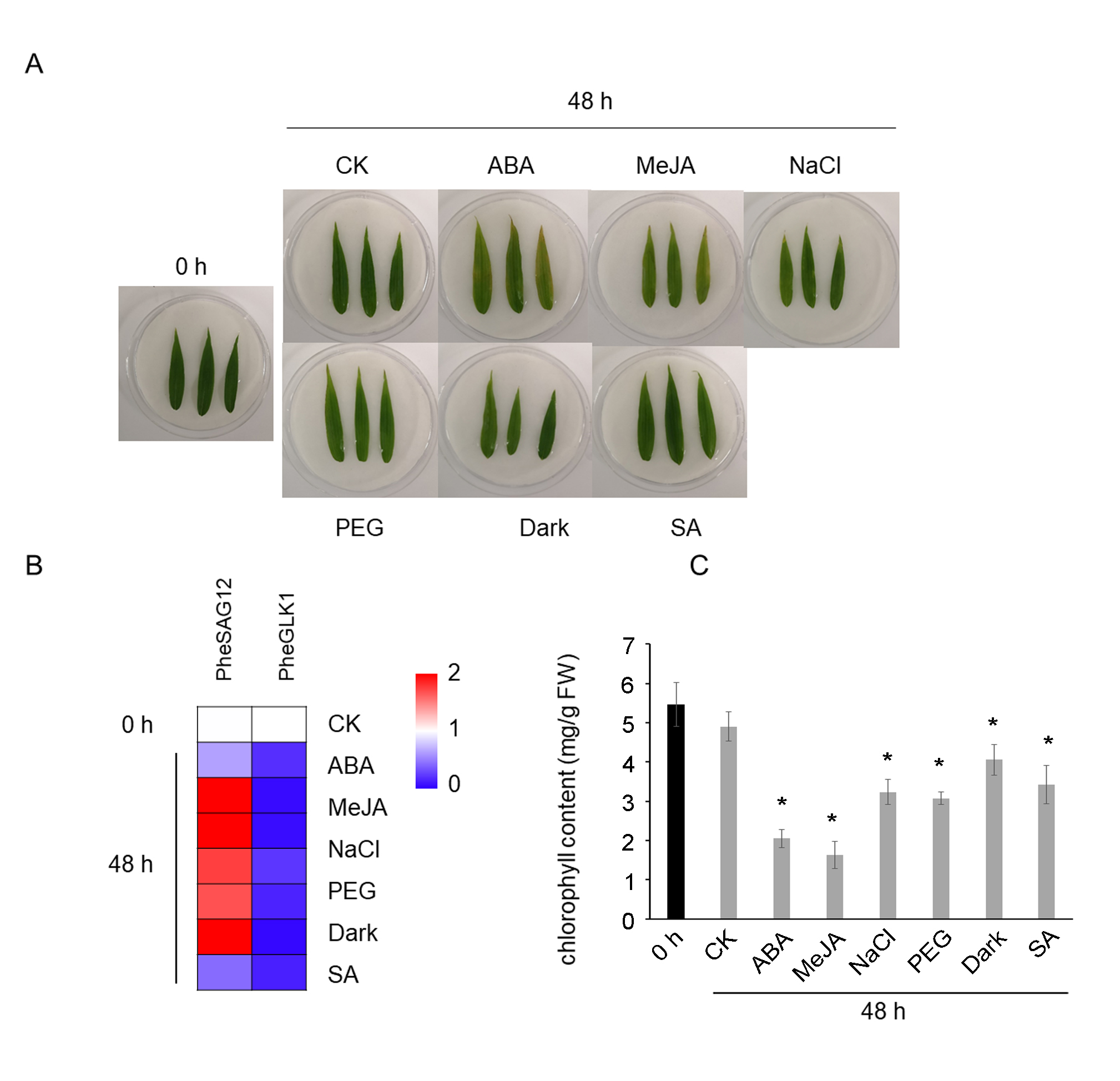

Supplement: Supplemental Information 5 — (A) Images of the leaves after 48 h of senescence-inducing treatments. (B) Heat map indicating the relative transcript ratio of PheGLK1 (PH01000738G0520) and PheSAG12 (PH01001461G0020) normalized against values from samples harvested 0 and 48 h after ethylene treatment, respectively. Values represent the mean from at least three biological replicates. Black color = not detected. Statistically significant differences compared with the corresponding controls (P < 0.05) are colored accordingly to the scale. (C) Chlorophyll content in leaf samples. Values represent the mean ± SE from at least three biological replicates. Statistically significant differences compared to not-treated control are marked with asterisks (P < 0.05). [file peerj-08-8716-s005.png]

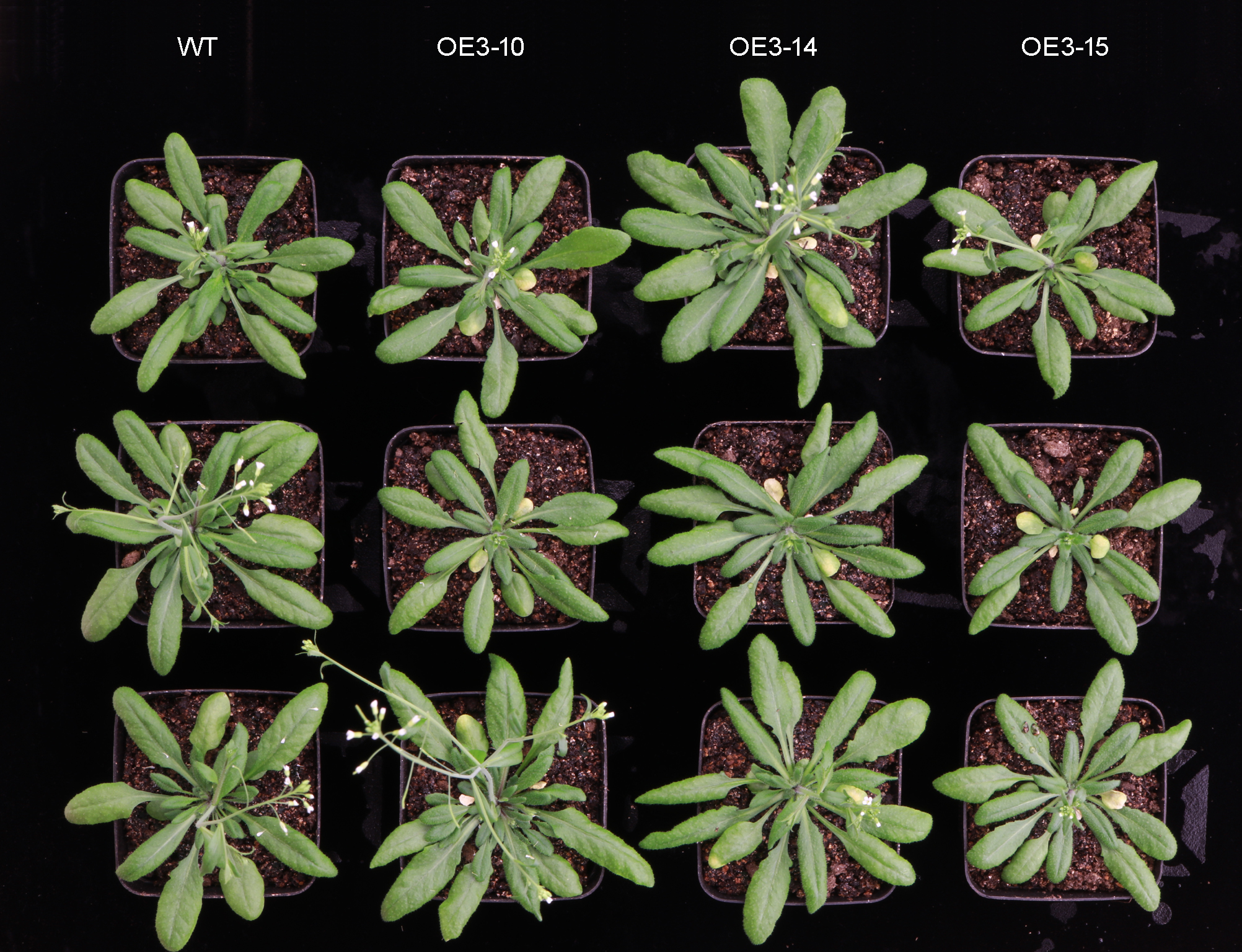

Supplement: Supplemental Information 6 — WT, wild-type Arabidopsis. OE3-10, OE3-14 and OE3-15, three independent lines. [file peerj-08-8716-s006.jpg]

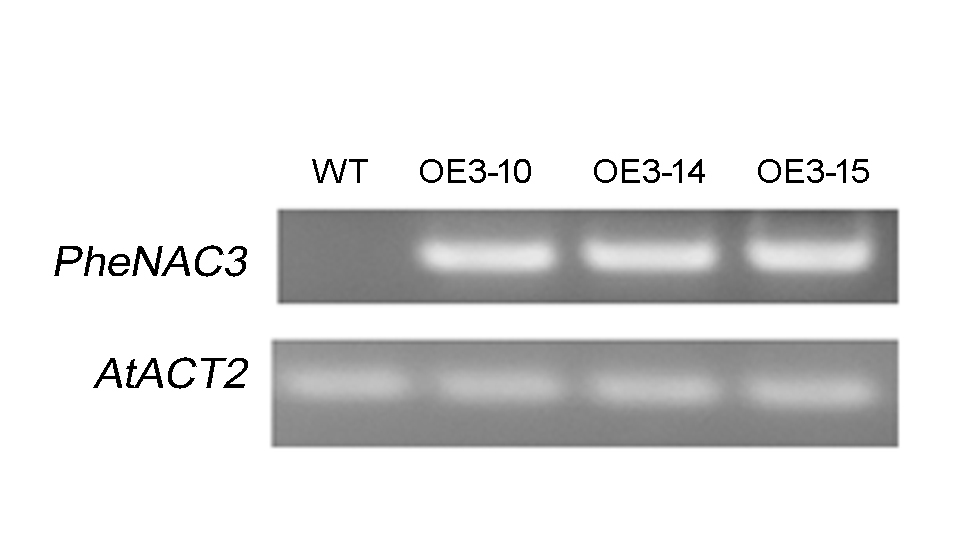

Supplement: Supplemental Information 7 — WT, wild-type Arabidopsis. OE3-10, OE3-14 and OE3-15, three independent lines. [file peerj-08-8716-s007.png]

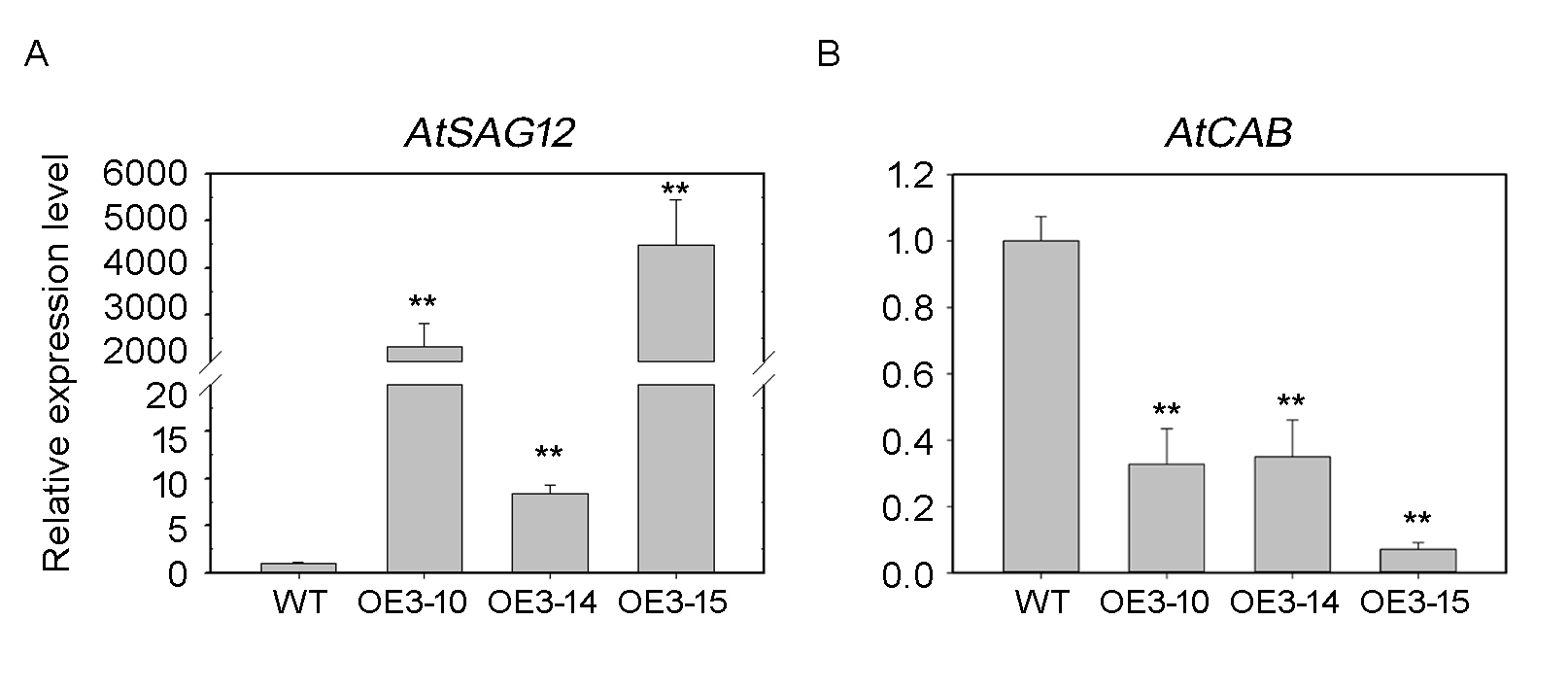

Supplement: Supplemental Information 8 — WT: wild-type Arabidopsis. OE3-10, OE3-14 and OE3-15: three independent lines. All experiments were repeated three times. Asterisks indicate the significant difference between WT and OE-PheNAC3 (*P < 0.05, **P < 0.01); error bars indicate the ± SE. [file peerj-08-8716-s008.png]

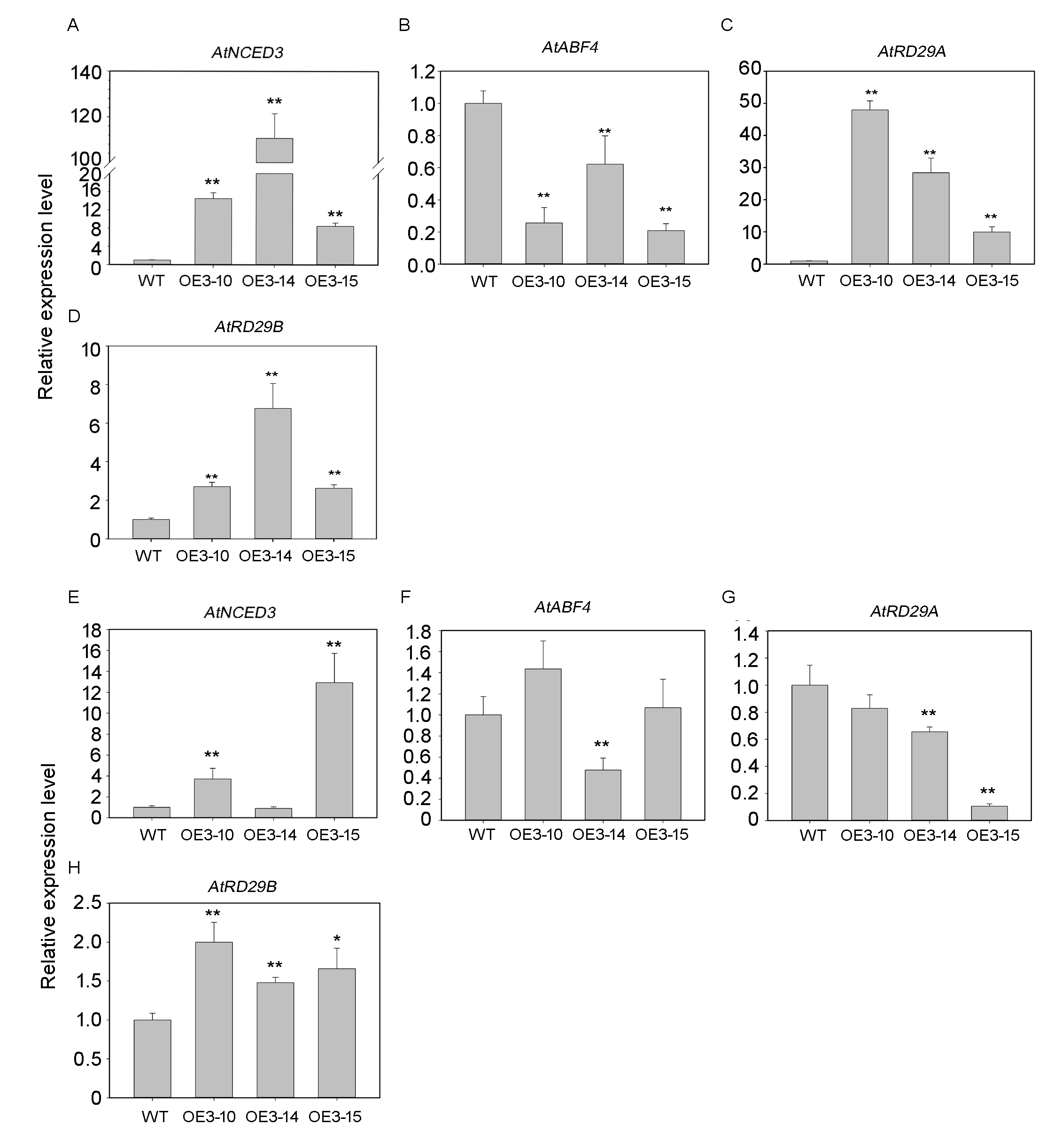

Supplement: Supplemental Information 9 — (A–D) The expression level of NCED3, ABF4, RD29A, and RD29B, in G2 of 5-week-old WT and OE-PheNAC3 plants. (E–H) the four genes in G3 of WT, wild-type Arabidopsis. OE3-10, OE3-14 and OE3-15, three independent lines. All experiments were repeated three times, with similar results. Asterisks indicate the significant difference between WT and OE-PheNAC3 (*P < 0.05, **P < 0.01); error bars indicate the ± SE. [file peerj-08-8716-s009.png]

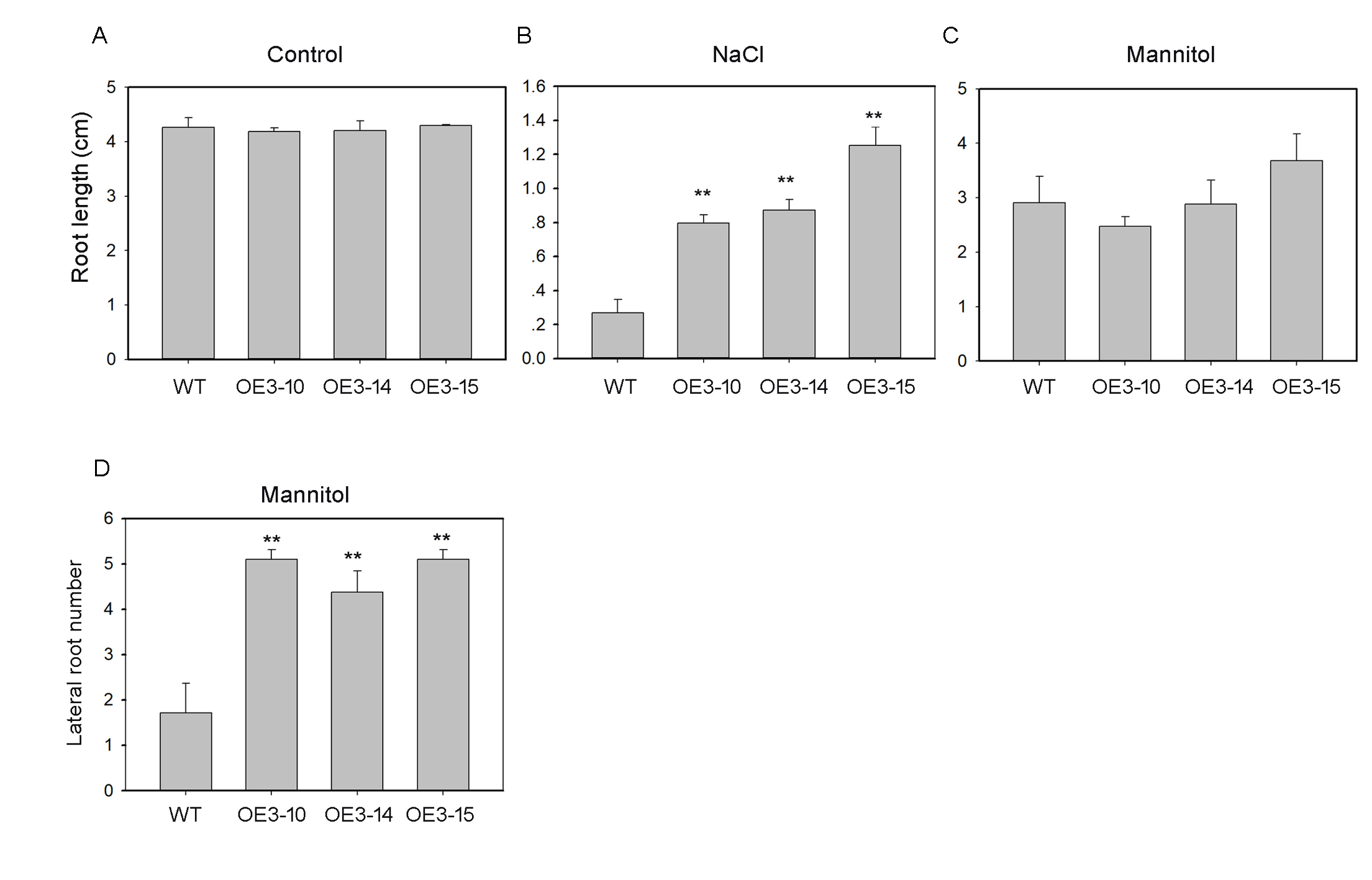

Supplement: Supplemental Information 10 — (A–C) Statistical analysis of taproot length of wild-type (WT) and PheNAC3-overexpressing Arabidopsis under mannitol or NaCl stress. (D) Statistical analysis of lateral root number of wild-type (WT) and PheNAC3-overexpressing Arabidopsis under mannitol. WT, wild-type Arabidopsis. OE3-10, OE3-14 and OE3-15, three independent lines. All experiments were repeated three times. Asterisks indicate the significant difference between WT and OE-PheNAC3 (*P < 0.05, **P < 0.01); error bars indicate the ± SE. [file peerj-08-8716-s010.png]

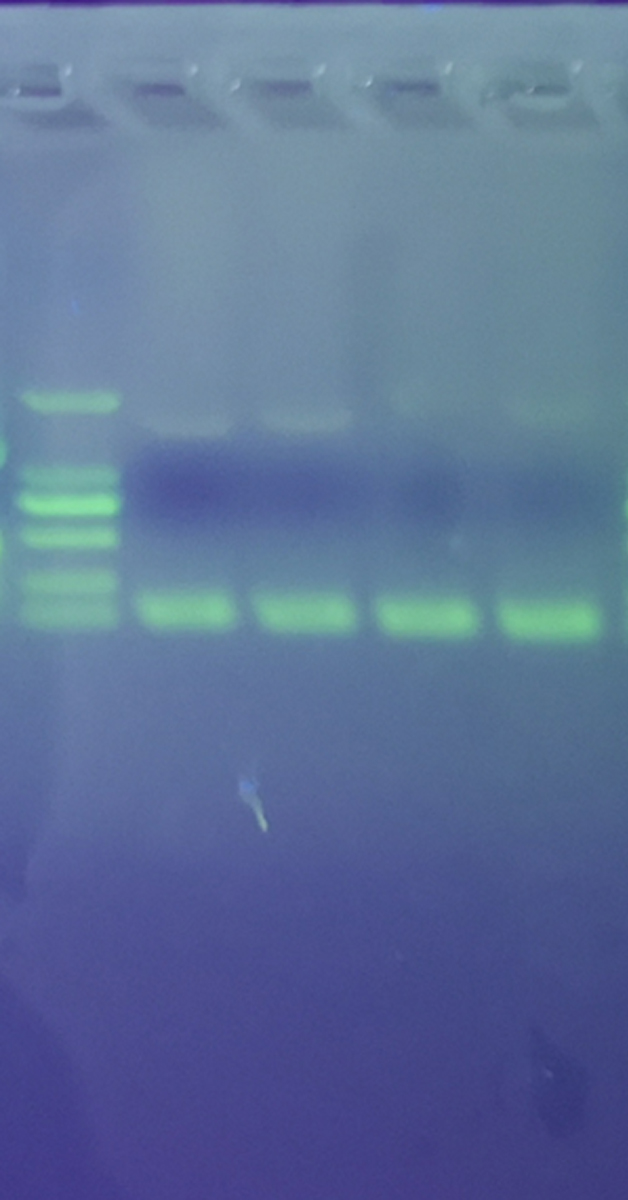

Supplement: Supplemental Information 13 [file peerj-08-8716-s013.zip › Raw data 1/Full-length_gels_and_blots/Actin2.jpg]

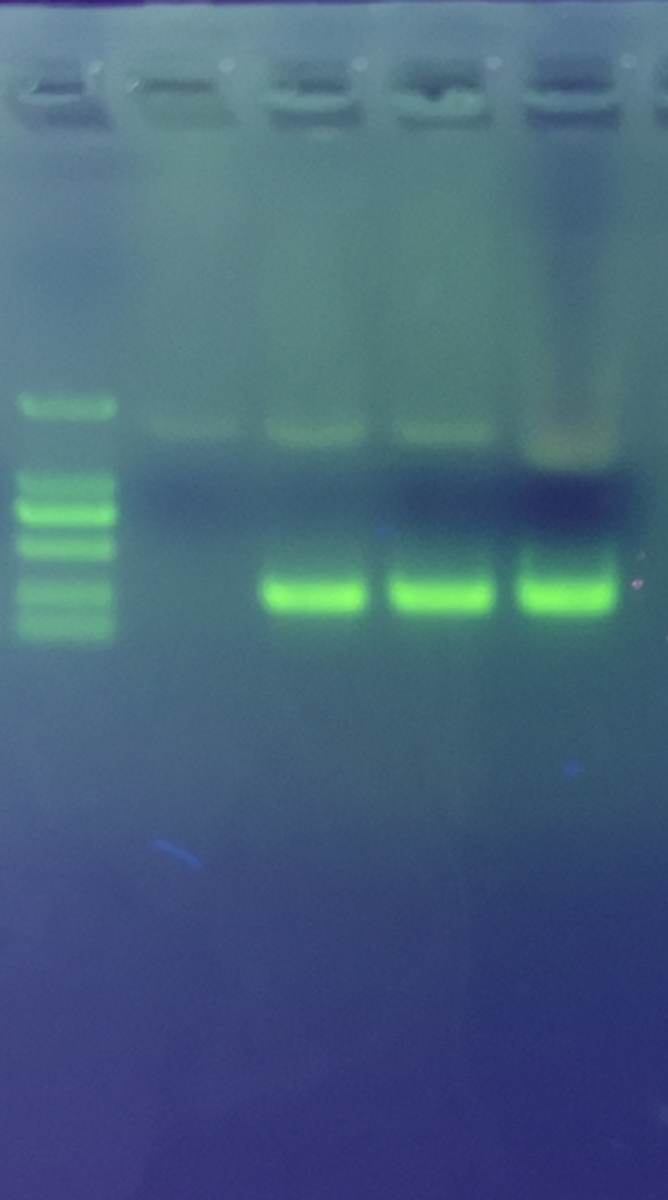

Supplement: Supplemental Information 13 [file peerj-08-8716-s013.zip › Raw data 1/Full-length_gels_and_blots/PheNAC3.jpg]
