# Supplementary material for: Overexpression of PheNAC3 from moso bamboo promotes leaf senescence and enhances abiotic stress tolerance in Arabidopsis
Source: PeerJ. 2020 Mar 31;8:e8716. doi: 10.7717/peerj.8716 (PMC7120055; doi:10.7717/peerj.8716)
Supplement: Supplemental Information 11 [file peerj-08-8716-s011.docx]

Table S1 The list for primers

| Name | Primer (5’ - 3’) |
| --- | --- |
| *PheNAC3* CDS sequence cloning | |
| CDS-PheNAC3-F | ATGAATTCCCTGAACATGGTGG |
| CDS-PheNAC3-R | TCAATCGAGTGAGTTCCACATCTGT |
| *PheNAC3* overexpression construct | |
| OE- PheNAC3-F | CGGGATCCATGAATTCCCTGAACATGGTGG |
| OE- PheNAC3-R | CCCAAGCTTTCAATCGAGTGAGTTCCACATCTGT |
| PheNAC3-GFP fusion vector | |
| 35S-EGFP- PheNAC3-F | GTTCATTTCATTTGGAGAGGACAGGATGAATTCCCTGAACATGGTGG |
| 35S-EGFP- PheNAC3-R | TCGACTCTAGAGGATCCCCGGGTATCGAGTGAGTTCCACATCTGT |
| GAL4 DBD fusion vector | |
| BD-PheNAC3-F | GGCCGAATTCCCGGGGATGAATTCCCTGAACATGGTGG |
| BD-PheNAC3-R | GCCGCTGCAGGTCGACGTCAATCGAGTGAGTTCCACATCTGT |
| qPCR primers | |
| RT-AtACT2-F | CGCTCTTTCTTTCCAAGCTC |
| RT-AtACT2-R | AACAGCCCTGGGAGCATC |
| RT-AtSAG12-F | TGGATACGGCGAATCTACTAACG |
| RT-AtSAG12-R | GCTTTCATGGCAAGACCACATAG |
| RT-AtCAB-F | CCAGAGGCATTCGCTGAGTTG |
| RT-AtCAB-R | CCTTACCAGTGACGATGGCTTG |
| RT-AtABF4-F | AACAACTTAGGAGGTGGTGGTC |
| RT-AtABF4-R | CTTCAGGAGTTCATCCATGTTC |
| RT-AtNCED3-F | GCTGCGGTTTCTGGGAGAT |
| RT-AtNCED3-R | GTCGGAGCTTTGAGAAGACGAT |
| RT-RD29A-F | TGGACACGAATTCTCCATCA |
| RT-RD29A-R | TTCCAGCTCAGCTCCTGATT |
| RT-RD29B-F | GGAGAGAGCAGAGAGGCTCA |
| RT-RD29B-R | CCGTTGACCACCGAGATAGT |
| RT-PheTip41-F | AAAATCATTGTAGGCCATTGTCG |
| RT-PheTip41-R | ACTAAATTAAGCCAGCGGGAGTG |
| RT-PheGLK1-F | CAGCACATATGCCGACTCAAG |
| RT-PheGLK1-R | GAATTGTGCATCTTGCTGGTTC |
| RT-PheSAG12-F | AACCATGGGCAGAGGAGTTG |
| RT-PheSAG12-R | ACAGCCGAGGAGATCTAGCA |
| RT-PheNAC3-F | CATCGATCGATCGATCAGCAA |
| RT-PheNAC3-R | CGACCTTGTTCAGGTCGACGT |
