# Supplementary material for: Overexpression of PheNAC3 from moso bamboo promotes leaf senescence and enhances abiotic stress tolerance in Arabidopsis
Source: PeerJ. 2020 Mar 31;8:e8716. doi: 10.7717/peerj.8716 (PMC7120055; doi:10.7717/peerj.8716)
Supplement: Supplemental Information 12 [file peerj-08-8716-s012.docx]

Table S2 Hormone and stress related cis-elements in promoters of *PheNAC3*

|  | Site Name | sequence | Numbers | function |
| --- | --- | --- | --- | --- |
| Hormone | ABRE | ACGTG, GCAACGTGTC, CGCACGTGTC, AACCCGG, | 7 | cis-acting element involved in the abscisic acid responsiveness |
|  |  |  |  |  |
|  |  |  |  |  |
|  |  |  |  |  |
|  |  |  |  |  |
|  |  |  |  |  |
|  |  |  |  |  |
|  |  |  |  |  |
|  | CGTCA-motif | CGTCA | 2 | cis-acting regulatory element involved in the MeJA-responsiveness |
|  |  | CGTCA |  |  |
|  | P-box | CCTTTTG | 2 | gibberellin-responsive element |
|  |  | CCTTTTG |  |  |
|  | GARE-motif | TCTGTTG | 1 |  |
|  | TGA-element | AACGAC | 1 | auxin-responsive element |
| Stress | LTR | CCGAAA | 1 | cis-acting element involved in low-temperature responsiveness |
|  | MBS | CAACTG | 1 | MYB binding site involved in drought-inducibility |
|  | STRE | AGGGG | 1 | activation by heat shock, osmotic stress, low pH, nutrient starvation |
|  | WRE3 | CCACCT | 2 | wound-response element 3 |
|  | WRE3 | CCACCT |  |  |
